# Sex-biased computations underlying differential set shift performance in mice

**DOI:** 10.1038/s41386-026-02397-z

**Published:** 2026-04-10

**Authors:** Nic Glewwe, Evan M. Dastin-van Rijn, Cathy S. Chen, Erin Giglio, Evan Knep, R. Becket Ebitz, Alik S. Widge, Nicola M. Grissom

**Affiliations:** 1 Department of Neuroscience, University of Minnesota, Minneapolis, MN USA; 2 Department of Biomedical Engineering, University of Minnesota, Minneapolis, MN USA; 3 Department of Psychiatry, University of Minnesota, Minneapolis, MN USA; 4 Department of Psychology, University of Minnesota, Minneapolis, MN USA; 5 Département de Neurosciences, Université de Montréal, Montreal, Canada

**Keywords:** Decision, Cognitive control

## Abstract

Cognitive flexibility can be defined as the ability to adaptively shift between choices or strategies based on environmental feedback. Flexibility is disrupted in numerous neuropsychiatric conditions. Individual differences in the computations supporting cognitive flexibility may reveal mechanisms of neuropsychiatric risk and resilience. One critical variable well known to influence individual differences in neuropsychiatric risk is sex. While previous research has identified sex differences in value based decision making in mice, whether sex reflects a major source of variation in cognitive flexibility remains unknown. To directly assess sex-biased individual differences in cognitive flexibility, we developed a novel touchscreen Set Shift task that permits robust and continuous testing in mice. Using this task, we discovered that female mice completed significantly more rule shifts with fewer errors than males. We next employed a suite of computational models that revealed sex-biased individual differences in the computations underlying cognitive flexibility. Our results suggest that following rule shifts, female mice learn the new rule faster and commit to exploiting rule choices sooner compared to males–sometimes because they follow multiple rules simultaneously. This suggests that increased choice stability in female rodents enhances commitment to a choice during periods of uncertainty and directly contributes to increased rule shifting. This supports the counterintuitive conclusion that a high degree of stable choice is a strong requirement for enhanced cognitive flexibility in the Set Shift task, one of the well-established cognitive flexibility tasks.

## Introduction

Cognitive flexibility is a key executive function, and can be defined as the ability to adaptively shift between choices or strategies based on environmental feedback. Maladaptive cognitive flexibility has been implicated in numerous neuropsychiatric conditions [1–6]. Understanding drivers of altered cognitive flexibility between individuals can thus be poised to reveal mechanisms of neuropsychiatric risk and resilience. An underappreciated feature of well-established cognitive flexibility tasks such as Attentional Set Shifting and Wisconsin Card Sorting Test is that strong performance requires *both* stability during periods of relative certainty *and* the ability to engage flexible decision making rapidly when the task environment changes [4, 7–11]. The need to balance stability with flexibility in value-based decision making can be framed as an explore-exploit tradeoff, suggesting that individual differences in explore-exploit balance may drive altered cognitive flexibility.

Multiple disorders involving cognitive flexibility show sex-biased prevalence [12–14]. Sex differences in cognitive flexibility are understudied, but significant sex differences have been repeatedly observed in the explore-exploit tradeoff. Female mice repeatedly execute rewarding choices and shift decision making strategies sooner following non-rewarded outcomes [12, 13, 15–18], leading to greater exploit behavior in females relative to males [15, 16]. These data suggest that sex differences in reward-guided decision making computations, including explore-exploit tradeoffs, may drive distinct approaches to cognitive flexibility tasks.

Designed to be homologous to human assays of cognitive flexibility, operant Attentional Set Shifting (Set Shift) tasks have been used to reveal neural dynamics important for attending to and shifting between attentional “sets” or rules within single sessions in rats and non-human primates [4, 10, 19, 20]. In rats, the operant Set Shift task has been used to assess how brain stimulation enhances cognitive flexibility, serving as a model for human psychiatric deep brain stimulation [4, 8, 11, 21]. Set shifting tasks for mice have been largely nonoperant, for example, the odor/digging medium discrimination task, and have revealed key roles for frontostriatal circuits in flexibility [9, 22–30] but lack temporal resolution. An operant set shifting task for mice that allows continuous rule shifts within a single session would permit direct translation of findings from mice to other species, providing the computational and temporal resolution benefits of a repeatable operant task with a method amenable to mouse models (e.g., future genetic manipulations and neuropsychopharmacological interventions).

We demonstrate a newly-developed operant touchscreen Set Shift task that permits robust and continuous testing in mice, and use this task to interrogate sex differences in the computations supporting cognitive flexibility. As in rat versions of this task [4, 10], the mouse Set Shift task employs rules across two dimensions (side and light) and allows repeated, automated, performance based rule shifts without requiring investigator intervention, permitting unlimited rule transitions (up to 200 daily trials), allowing computational modeling of behavioral strategies. We discovered that female mice persistently completed significantly more rule shifts with fewer errors than males. Using a combined reinforcement learning-drift diffusion model [4] we determined that both choice pre-commitment (bias) and value updating processes (learning rate) are enhanced in female mice. To measure the explore-exploit tradeoff, we used an input-output hidden Markov model (ioHMM), and determined that females more rapidly exit exploration and commit to exploiting rule states sooner than males. Female mice were sometimes more likely to exploit multiple rules simultaneously, and this was associated with the highest success rates. These results suggest that enhanced exploit behavior, shown as commitment to (one or more) rules, promotes enhanced set shifting.

## Methods

### Animals

Thirty-two BL6129SF1/J mice (16 F, 16 M) were obtained from Jackson Laboratories (stock #101043). Animal counts were determined by power analysis (see below). Animals were food restricted to 85–95% of free feeding bodyweight, with *ad libitum* access to water. Animals were pre-exposed to the reinforcer (50% water diluted vanilla Ensure) in the home cage. Starting at 12 weeks of age, behavioral training and testing in the operant chambers occurred five days per week (Monday-Friday), and animals were fed each day following behavior. Behavioral training and testing occurred in the same chamber for each animal and operant chambers were located in unique female and male behavior rooms to avoid potential confounds. Animals were housed in 12 h reversed light cycle housing to permit operant testing during the dark period, beginning at 0800 h. One female animal unexpectedly died before Set Shift training began due to natural causes, producing a final n of 31 mice (15 F, 16 M). All animals were cared for in accordance with the National Institutes of Health and the University of Minnesota Institutional Animal Care and Use Committee (IACUC) guidelines, and all procedures were IACUC reviewed and approved.

### Apparatus

Sixteen triangular touchscreen operant chambers (Bussey-Saksida design, Lafayette Instrument Co., Lafayette, IN), enclosed inside sound attenuating cabinets were used for behavioral training and testing. Two of the three chamber walls were black, acrylic plastic. The third wall housed the touchscreen and was positioned directly opposite of the magazine. A two-hole mask over the touchscreen defined two possible choice options. The magazine provided Ensure liquid reinforcer (280 ms pump duration, ~7ul). ABET-Cognition software (Lafayette Instrument Co., Lafayette, IN) was used to program operant behavioral schedules and to collect all training and testing data.

### Pre-training

After habituation to the operant chamber, animals were exposed to daily touchscreen training schedules including Initial Touch, Must Touch, and Must Initiate as previously described [15]. Animals completed the Punish Incorrect schedule but the stimuli displayed on the touchscreen during the task were adapted to be consistent with stimuli presented during the Set Shift task. After mice reached criterion on each schedule (two consecutive days with 30 trials completed in 30 min or two consecutive days with 60 trials completed in 60 min, depending on schedule), they moved on to the next schedule. On average, animals spent five days on each schedule.

### Rule shaping

To control for training order effects, animals were assigned to learn either the “light” rule (*n* = 16, 8 F, 8 M), the “right” rule (*n* = 7, 3 F, 4 M), or the “left” rule (*n* = 8, 4 F, 4 M) first, ensuring that cagemates of each sex were evenly distributed across conditions (Supplementary Fig. 1a). Rule shaping schedules were rewarded deterministically. During Light Shaping, animals only received reward for choosing the “light” image (illuminated square), while during Left and Right Shaping, animals were only rewarded for choosing the left option or the right option, respectively. To meet the criterion for each schedule, animals needed to perform at least 60 trials in 60 min (maximum of 200 trials or 60 min allowed) and complete at least 10 consecutive correct choices for two consecutive days. Animals spent five days on each schedule, and each animal reached the shaping performance criterion (Supplementary Fig. 1c), confirming that all animals learned each individual rule (see additional details in Supplementary Methods).

### Touchscreen set shift task for mice

**Fig. 1:**
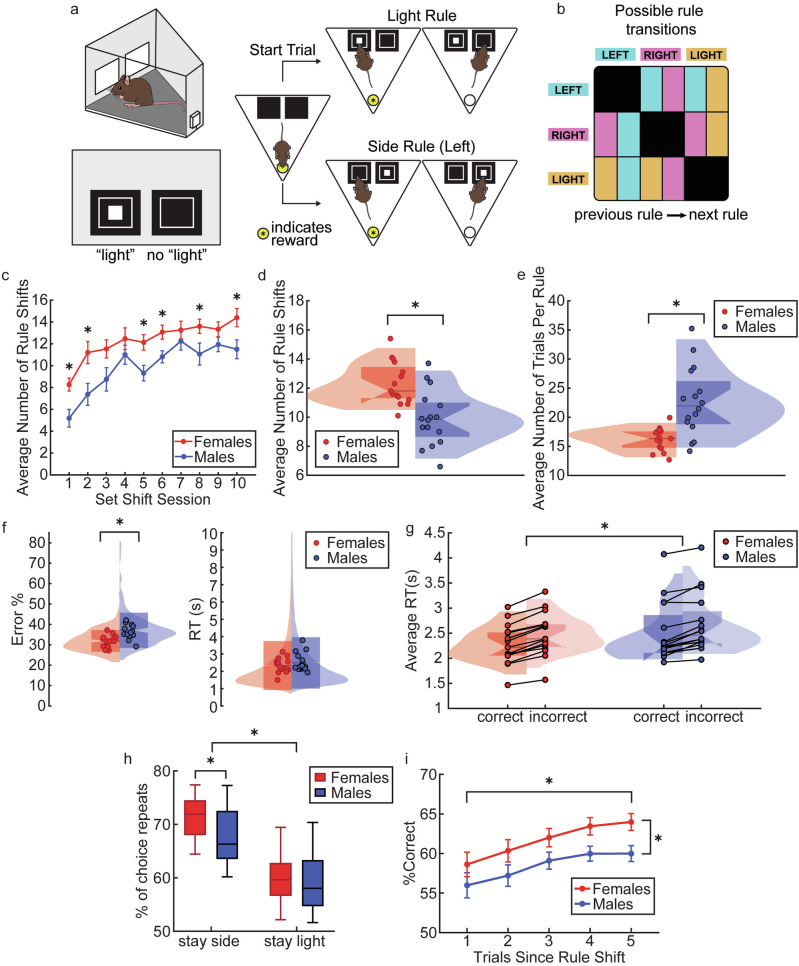
Female mice show enhanced Set Shifting compared to males. **a** The mouse operant touchscreen Set Shift task. In the images displayed on the touchscreen, the outer white square indicates that the touch/choice is active while the inner white illuminated square is the “light” cue. During the light rule, selection of the light cue is rewarded regardless of what side it appears on. In the side rules (e.g. left rule as pictured), selection of one side (e.g., left) is rewarded regardless of whether or not the light cue appears on that side. After five consecutive correct choices without omissions in a given rule, the rule shifts. **b** All possible rule transitions. Rules are selected in a random equal-number way. **c** Though Set Shift performance (number of completed rule shifts) improves for all animals across the 10 total days on Set Shift, female mice consistently complete more average rule shifts compared to males across daily sessions (*n* = 31, 15 F/16 M, ~200 trials/session, 10 sessions/animal, GLMM main effect of sex: *p* = 0.0004, β1 = –3.1333; main effect of session: *p* = 5.2951e–09, β2 = 0.5079; sex*session interaction: *p* = 0.2631, β3 = 0.1319). **d** Averaging across Set Shift days, female mice complete significantly more rule shifts than males (*n* = 31, 15 F/16 M, ~2000 trials/mouse; two-sample *t* test: *p* = 5.2220e–04, effect size  = 2.4079). **e** Female mice reach criterion (5 consecutive correct choices without omissions) in fewer trials than males on average, spending less time in each rule and rule shifting faster than males (two-sample *t* test: *p* = 3.8835e–04, effect size = –6.4282). **f** Female mice demonstrate fewer errors than males during Set Shift (GLMM main effect of sex: *p* = 6.9163e–05, β1 = 0.22976) but there are no significant sex differences in overall, average response time (RT) (GLMM main effect of sex: *p* = 0.4828, β1 = –0.1282). **g** Considering how RT differs by performance across sex reveals a significant main effect of performance (correct/incorrect) (GLMM main effect of performance: *p* = 7.2609e–262, β2 = –0.7381) and a marginally significant interaction effect between sex and performance (sex*performance interaction, *p* = 0.0534, β3 = –0.0588). **h** Comparing how frequently animals repeated previously correct side versus light choices (regardless of the current rule), we found that animals repeat previously correct side choices more often than they repeat previously correct light choices (two-way repeated measures ANOVA: *p* = 1.34012e–11, F(1,58) = 70.47). On average, female mice repeated the previously correct side choice even more frequently than males (two-sample *t* test: *p* = 0.0364, effect size = –3.7211), demonstrating enhanced correct choice repetition. **i** All animals get more choices correct as trials increase following a rule shift, but female mice make significantly more correct choices across trials following rule shifts compared to males (two-way repeated measures ANOVA effect of trial: *p* = 0.0006, F(4,145) = 5.165; effect of sex: *p* = 6.47705e–05, F(1,145) = 16.93). Figure 1c–i depict mean and SEM, Fig. 1d–g depict median (solid line), 1–99th percentile of data in maximum shaded area vertically and kernel density horizontally, and inner hourglass shaded area depicts 25–75% confidence interval of data, and Fig. 1h depicts median (solid line) as well as min to max (whiskers).

The touchscreen operant Set Shift task (Fig. 1a) was modified from standard operant chamber designs in de Oliveira et al. (2021) and Reimer et al. (2024) and originally developed by Darrah et al. 2008 [4, 10, 31]. To probe cognitive flexibility, the Set Shift task employs two different rules–a light rule where the “light” (illuminated square) is rewarded regardless of what side it appears on, and a side rule where a side (left/right) is rewarded regardless of the light location (Fig. 1a). If a choice is not made within the response time limit, an omission is logged. Following five consecutive correct choices without omission in a given rule, the criterion is met and the rewarded rule shifts to a different rule without warning. The lack of reward for a previous rule response is the only indication that the rewarded rule has shifted. During Set Shift, animals receive reward by inhibiting a prepotent response/cease exploiting the recent past rule, and learn the new rule after the environment “shifts” via exploration. Unique to this Set Shift task, the rewarded rule shifts to any of the other rules (six total transition combinations) (Fig. 1b), allowing for intra- and extradimensional shifts. The next rule is selected in a random-equal-number way to prevent predictability. Trials are initiated via nose-poke at the magazine when the tray light turns on. Following initiation, animals have a response time limit (10-3 s depending on schedule) during which they must execute a choice before a 3 s timeout begins. Incorrect choices similarly evoke this timeout, paired with an incorrect tone (3000 Hz). Immediately following a correct choice, the magazine distributes liquid reward and the tray light turns on. Once reward is collected, the tray light turns off. After each choice or omission, animals can initiate the next trial after a 3 s inter-trial interval (ITI). The Set Shift task lasts for 200 trials or 60 minutes. Mice are allowed to complete as many rule shifts as they can in the allotted time. The baseline Set Shift task allowed a 10 s response time limit. The response time limit exists to avoid periods where the animal is inactive in the chamber without the trial resetting. This concept is also utilized in human cognitive flexibility tasks [8, 11]. To assess the ability of mice to perform the task with the same response time limit as rats in a standard operant chamber [4, 10], a second Set Shift schedule was created with a response time limit of 3 s (see Supplementary Methods).

### Data analysis

Analyses were conducted using MATLAB version 2022b, Python version 3.11, and GraphPad Prism 10. Violin plots were created using the al_goodplot function in MATLAB [32]. Generalized linear mixed effects models (GLMM), repeated measures analysis of variance (ANOVA), and two-sample *t* tests were used for statistical testing. Animal counts were predetermined by a power analysis to detect the standard effect size of sex differences common in previous publications from the lab [15, 16], targeting 80% power (G*Power 3.1).

Consistent with previous studies [4], repeated-measures data including response times, accuracy, and number of completed rule shifts were analyzed with GLMMs as gamma regressions for response times, logistic regressions for errors/performance, and linear regressions for completed rule shifts. The general model for these analyses:

$${{\rm{DependentVariable}}} \sim {{\rm{Sex}}}+{{\rm{Session}}}+(1|{{\rm{Subject}}})$$

Across analyses, statistical significance was determined if *p* < 0.05 and error bars represent standard error of the mean.

### Reinforcement learning drift diffusion model (RLDDM)

**Fig. 2:**
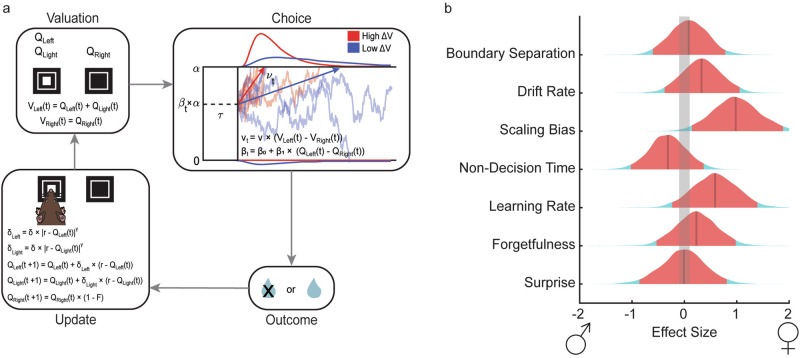
Stronger effects of choice bias and learning rate in female mice. **a** Schematic of the reinforcement learning drift diffusion model (RLDDM). Values are assigned to each choice based on estimates of the current side (left and right) and “light” cue values. The model then makes a choice using a drift diffusion process informed by the difference in total value. The distributions within the Choice box illustrate the effect of faster (red) versus slower (blue) drift rates–trials with higher value differences lead to faster response times and more consistent choices. After the model makes a choice, values are updated by a reinforcement learning process based on the outcome of the previous choice. **b** Distributions of the effect of sex on each model parameter across 4000 posterior draws. The 95% highest density interval is depicted in red/orange, the solid line indicates the median of the distribution for each parameter, and the shaded gray area on either side of 0 represents the region of practical equivalence (ROPE) for a null effect (effect size <0.1). Effect size by sex is indicated by the x-axis, with 2 representing a stronger effect in females and −2 representing a stronger effect in males. Effect size is defined as Parameter_female minus Parameter_male divided by parameter variance. Distribution details for all parameters are included in Supplementary Materials (Table S1).

We used a reinforcement learning drift diffusion model (RLDDM) to model decision making during Set Shift sessions 5–10, following initial learning of the full task [4]. This model combines elements of two prominent computational modeling approaches for decision making: (1) a reinforcement learning model, which updates trial-to-trial value learning, and (2) a drift diffusion model, which models evidence accumulation up to choice. The RLDDM was fit to mouse Set Shift behavior (the sequences of choices and response times each session) using Markov-Chain Monte Carlo with the HDDM Python package [4, 33]. To ensure reliable estimation of parameters, four independent chains (2000 total samples) were run for each model and convergence was confirmed by assessing whether the Gelman-Rubin statistic was <1.1 [4, 34]. To examine sex-based influence, we allowed the model to fit different intercept terms for males and females (see additional details in Supplementary Methods). This allows us to evaluate how strongly each model parameter (boundary separation, drift rate, bias, non-decision time, learning rate, forgetfulness, and surprise) is affected by sex. Using the RLDDM, values are assigned to each choice based on estimates of the current side (left/right) and “light” values. The model uses those values to generate a choice using a drift diffusion process, accumulating evidence until the decision boundary is reached. Trials with a larger total difference in value between choices are more likely to have shorter response times and more consistent choices. Depending on whether a choice is rewarded, values are updated using a reinforcement learning process (Fig. 2a). Modeling the data from each group (females/males), the posterior distribution of group model parameter differences inform which computations show the largest sex biases. The full set of equations for the RLDDM:

The value of a choice (V) is the sum of its respective side and light values (Q):

$$V({side},t)=Q({side},t)+({Light}(t)=={side})\times Q({light},t)$$

Drift ($v(t)$) on each trial is directed proportionally to the side with the highest value:

$$v(t)={v}_{0}\times (V({Left},t)-V({Right},t))$$

Bias ($\beta (t)$) on each trial is directed proportionally to the side with the highest value, ignoring the light:

$$\beta (t)=\phi \left({\beta }_{0}\right.+{\beta }_{1}\times (Q({Left},t)-Q({Right},t))$$

On each trial, non-decision time ($\tau (t)$) was sampled from a uniform distribution with mean ${\tau }_{0}$ and width ${\tau }_{{\mathrm{var}}}$:

$$\tau (t) \sim {Uniform}({\tau }_{0},{\tau }_{{\mathrm{var}}})$$

For each trial, response times (RT) follow a Wiener First Passage Time (WFPT) distribution with parameters for drift ($v(t)$), boundary separation ($\alpha$), bias ($\beta (t)$), and non-decision time ($\tau (t)$):

$${RT}(t) \sim {WFPT}(v(t),\alpha ,\beta (t),\tau (t))$$

Separate non-linear learning rates ($\delta$) were calculated for each chosen feature ($C$) to model surprise ($\gamma$):

$$\delta (C,t)={{\delta }_{0}{|r}\left(t\right)-Q\left(C\right)|}^{\gamma }$$

The value of each chosen feature was updated via a standard Rescorla-Wagner learning rule:

$$Q(C,t+1)=\delta (C,t)\times (r(t)-Q(C,t))$$

The value of unchosen features diminishes according to a forgetfulness factor ($F$):

$$Q(C,t+1)=Q(C,t)\times (1-F)$$

The RLDDM has 11 parameters in total: initial values for the side and light features, drift rate $({v}_{0})$, baseline bias (${\beta }_{0}$), scaling bias (${\beta }_{1}$), non-decision time (${\tau }_{0}$), variability of non-decision time (${\tau }_{{\mathrm{var}}}$), boundary separation ($\alpha$), baseline learning rate (${\delta }_{0}$), surprise ($\gamma$), and forgetfulness ($F$). Parameters were estimated using a Bayesian hierarchical approach. To determine the effect of sex on model parameters, the group-level distributions for the parameters were assessed using the probability of direction (PD) and the region of practical equivalence (ROPE) [4, 35]. PD refers to the proportion of the parameter distribution greater than 0 with a value above 0.5 (indicating a general increase in the parameter across groups) and a value below 0.5 (indicating a general decrease in the parameter across groups). ROPE refers to the proportion of the parameter distribution that falls within a region that is practically equivalent to a null-effect (+/- 0.1). PD indicates the existence and direction of an effect while ROPE establishes significance.

### Input-output hidden Markov model (ioHMM)

**Fig. 3:**
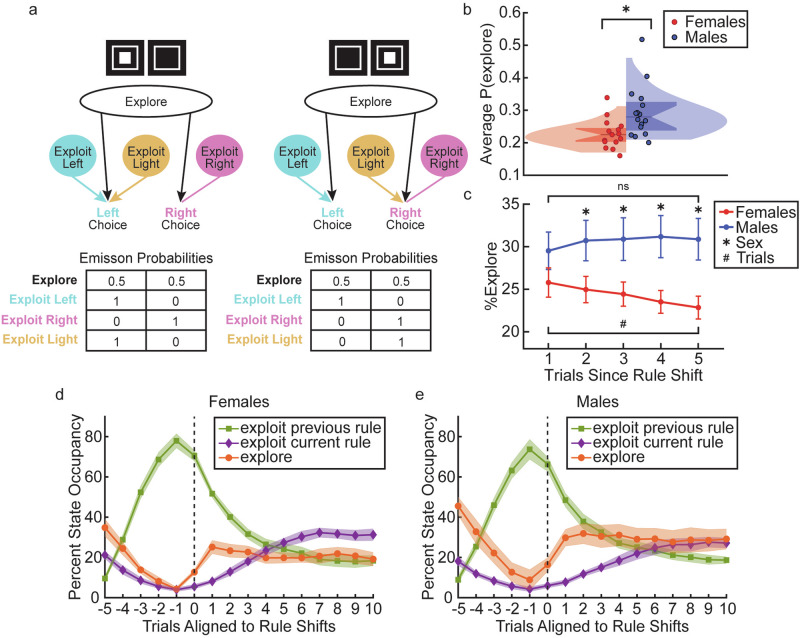
Faster transitions out of exploration following rule shifts in females. **a** Schematic of the input-output hidden Markov model (ioHMM), illustrating how the emission probabilities for making a left or right choice in each state change in response to the input (location of the light stimulus). **b** On average, male mice have a higher probability of being in the explore state compared to females throughout the Set Shift task (two-sample *t*-test: *p* = 0.0126, effect size = –0.0666). **c** One to five trials after rule shifts, there is an overall effect of sex, but not trial since rule shift, on the percent of explore labeled trials (two-way repeated measures ANOVA effect of sex: *p* = 2.19611e–06, F(1,145) = 24.33; effect of trials: *p* = 0.9906, F(4,145) = 0.0715). However, the average percent of explore labeled trials significantly decreases in female mice (two-sample *t* test: *p* = 0.0432, effect size = −2.9487), while male mice remain in the explore state across trials following rule shifts, presenting a sex difference in the percent of explore labeled trials for trials 2–5 after rule shifts (two-sample *t* test comparison of female and male %Explore one trial after rule shifts: *p* = 0.1967; two trials after rule shifts: *p* = 0.0546; three trials after rule shifts: *p* = 0.0359; four trials after rule shifts: *p* = 0.0123; and five trials after rule shifts: *p* = 0.0084). Average percent of trials in each of three states–explore, exploit previous rule, and exploit current rule–five trials before and 10 trials after rule shifts (trial 0) for females (**d**) and males (**e**). Exploit previous rule refers to the rule that was in effect prior to the rule shift at trial 0 (trials -5 through -1) while exploit current rule refers to the active rule following the rule shift at trial 0 (trials 0–10). The black dotted line (0) indicates when the rule shifts. Trial 0 is the first trial in the new rule and represents that 0 trials have occurred in the new rule. On average, all mice similarly exploited the previous rule prior to rule shifts and subsequently transitioned away from exploiting the previous rule following rule shifts (exploit previous rule state occupancy GLMM main effect of sex: *p* = 0.4285, β1 = –1.306; main effect of trial: *p* = 1.1011e–26, β2 = –2.028). Consistent with panels (**b** and **c**), the average percent of explore labeled trials throughout this window significantly differed by sex (explore state occupancy GLMM main effect of sex: *p* = 0.0035, β1 = 7.6856; main effect of trial: *p* = 0.1690, β2 = 0.1195). On average, female mice transitioned to exploiting the current rule state within 10 trials following rule shifts while males did not (exploit current rule state occupancy GLMM main effect of sex: *p* = 0.0003, β1 = –2.709; main effect of trial: *p* = 1.5114e–82, β2 = 1.6112). Figure 3b depicts median (solid line), 1–99th percentile of data in maximum shaded area vertically and kernel density horizontally, and inner hourglass shaded area depicts 25–75% confidence interval of data. Figure 3c–e depicts mean and SEM.

An input-output hidden Markov model (ioHMM) was used to label latent cognitive states underlying Set Shift behavior. The HMM framework assumes that choices are generated from some unobserved latent cognitive states. Our model identified two general types of cognitive states–exploration and exploitation–defined by their unique patterns of choice. Here, choice behaviors are modeled as emissions (observations) from one of the four distinct latent cognitive states–explore, exploit left, exploit right, and exploit light (Fig. 3a). In the explore state, the emission probability for specific choice types are uniform, meaning that the generation of all choice types (left/right/light) are equally probable. The exploit states only emit the type of choice being exploited (e.g., the exploit left state only generates left choices). The transition matrix fit to the subject captures the unique transition probability between these four states in each individual. To disambiguate between choice dimensions (side/light), the location of the light cue was used as an input layer into the ioHMM. Together, the final ioHMM receives each animals’ choice sequences and the respective locations of the light cue, and outputs the most probable latent state for each trial.

The base ioHMM MATLAB code was adapted from Ebitz et al., 2019, Ebitz et al., 2020 [19, 20], and Kevin Murphy’s MATLAB HMM toolbox. A detailed description of the ioHMM:

Within the HMM framework, choices or “emissions” (y) are generated by an unobserved decision process within some hidden, latent state (z). These latent states are defined by both the probability of making each choice (k) out of (N_k_) possible options given the state of the system (e.g., location of the light) and the probability of transitioning from each state to every other state [36]. During exploration, animals have an equal, but random probability of making left and right choices regardless of the light location.

$$p({y}_{t}={k|}{z}_{t}={explore})=\frac{1}{{N}_{k}}$$

However, the location of the light is critical for disambiguating between the different rule states. For example, if an animal is following the left rule, they will repeatedly make left choices regardless of what side the light is on. If an animal is exploiting the light rule, they will only make left choices when the light is on the left side and right choices when the light is on the right side. The observation model for rule states was dependent on whether or not the observation (choice + light location) met the condition for the current rule:

$$p({y}_{t}={k|}{z}_{t}={rul}{e}_{i},k \in {rul}{e}_{i})=1$$

$$p({y}_{t}={k|}{z}_{t}={rul}{e}_{i},k \, \notin \, {rul}{e}_{i})=0$$

The latent states in this model are Markovian, meaning that they depend only on the most recent state (z_t_) and the most recent location of the light (l_t_), and are independent of time:

$$P({z}_{t}|{z}_{t-1},{l}_{t-1},{y}_{t-1},...,{z}_{1},{l}_{1},{y}_{1})=P({z}_{t}|{z}_{t-1},{l}_{t-1})$$

The probabilities of each state transition were described by the one-time-step probability of transitioning between every combination of past and future states (*i*,*j*). Unlike the emissions matrix, the transition matrix was not influenced by the location of the light.

$$p({z}_{t}={i|}{z}_{t-1}=j)$$

The model had four possible states (one explore state and three exploit (rule) states). Parameters were tied across exploit states such that each exploit state had the same probability of beginning from exploration and of sustaining itself. Transitions from exploration to the exploitative states were similarly tied, making it equally likely to start exploiting any of the three rule states after exploration. Transitions directly between exploit states were not permitted, and choices were initialized in the explore state. The model assumed that exploration was required in order to gain enough information to start exploiting a new rule, even if only for a single trial. This modeling choice reduced the number of free parameters to estimate, allowing us to estimate fewer parameters more accurately with a limited number of trials. As such, the model had two free parameters.

The model was fit via expectation-maximization using the Baum-Welch algorithm [37]. This algorithm identifies a (possibly local) maxima of the complete-data likelihood based on the joint probability of the latent state sequence and the sequence of observed choices. The algorithm was reinitialized with random seeds 20 times, and the model that maximized the observed (incomplete) data log likelihood was selected as the best for each session. To label latent states from choices, the Viterbi algorithm was used to identify the most probable *a posteriori* sequence of latent states [38, 39].

## Results

### Female mice show enhanced set shifting compared to males

All animals (BL6129SF1/J, *n* = 31, 15 F, 16 M) successfully learned to perform the Set Shift task (Fig. 1a, b) and completed within-session rule shifts, regardless of training order (Supplementary Fig. 1b). Following exposure to each rule individually, testing began on the full Set Shift task including all three rules. A robust sex-biased difference in the number of rule shifts completed emerged on the first day of the full Set Shift task (Fig. 1c). Training order also influenced the number of rule shifts completed on the first day of Set Shift. Animals that completed side shaping last (immediately preceding the full Set Shift task) performed significantly fewer rule shifts compared to animals that completed light shaping last (Supplementary Fig. 1c,d). This early training order effect interacted with sex differences. Male mice that completed side shaping last performed the fewest number of rule shifts on the first day of Set Shift (Supplementary Fig. 1e) (two-way repeated measures ANOVA effect of training order (last rule): *p* = 0.0022, F(2,25) = 7.883; effect of sex: *p* = 0.0004, F(1,25) = 16.50). Despite the initial presence of these training order effects, they did not persist past the first day on the full Set Shift task.

Female mice consistently completed more rule shifts on average compared to males, though all animals’ Set Shift performance improved across 10 days on the task (Fig. 1c) (*n* = 31, 15 F/16 M, ~200 trials/session, 10 sessions/animal, GLMM main effect of sex: *p* = 0.0004, β1 = –3.1333; main effect of session: *p* = 5.2951e–09, β2 = 0.5079; sex*session interaction: *p* = 0.2631, β3 = 0.1319). This was not explained by other behavioral differences as the number of completed trials, session length, response latencies, and relationship between body weight and performance were not significantly different across sexes (Supplementary Figs. 2, 3). To further examine this main effect of sex, we collated Set Shift performance across 10 days. Averaging across sessions reveals robust sex-biased individual differences in rule shifting performance where female mice complete more rule shifts (*n* = 31, 15 F/16 M, ~2000 trials/mouse; two-sample *t* test: *p* = 5.2220e–04, effect size = 2.4079), meeting criterion to shift in fewer trials than males (two-sample *t* test: *p* = 3.8835e–04, effect size = –6.4282) (Fig. 1d, e). Limiting analyses to the later Set Shift sessions produced similar results (Supplementary Fig. 4), indicating that the contributing factors related to these sex differences were not transient.

Multiple factors contribute to the number of rule shifts animals can perform. Female mice may complete more rule shifts than males because they are more accurate during the task, or because female mice are simply faster at the task. To test these two hypotheses, we assessed average accuracy and response times (RT) across sessions and identified that female mice make fewer errors than males during Set Shift (GLMM main effect of sex: *p* = 6.9163e–05, β1 = 0.22976), with no significant differences in overall average RT (GLMM main effect of sex: p = 0.4828, β1 = –0.1282) (Fig. 1f). Though RT does not differ across sex alone, average RT is sensitive to performance such that RTs during correct trials were significantly faster than RTs during incorrect trials at both the individual and group levels (GLMM main effect of performance: *p* = 7.2609e–262, β2 = –0.7381) however, this effect was stronger in the female mice (sex*performance interaction, *p* = 0.0534, β3 = –0.0588) (Fig. 1g). This suggests that sex-biased individual differences in the number of completed rule shifts emerge not simply due to females completing trials faster than males, but due to differences in trial accuracy.

Previous research has identified that female rodents demonstrate both higher choice stability [15, 16] and greater reward sensitivity [12, 13, 15–18] compared to males. One possibility is that females might be more able to use reward as a signal to repeat correct choices more frequently. Consistent with these concepts, not only did female mice make more correct choices than males during the Set Shift task, they used trial feedback (reward/no reward) differently. Though all animals more frequently repeated the previously correct side choice than the previously correct light choice (two-way repeated measures ANOVA: *p* = 1.34012e–11, F(1,58) = 70.47), females demonstrated significantly higher correct choice repetition compared to males (two-sample *t* test: *p* = 0.0364, effect size = –3.7211) (Fig. 1h). This had the effect of leading to increased female performance in shifts that were both intra- and extra-dimensional (Supplementary Fig. 5). Within the first five trials following a rule shift, accuracy increases across trials in all animals, but female mice are consistently more accurate across these post-shift trials (two-way repeated measures ANOVA effect of trial: *p* = 0.0006, F(4,145) = 5.165; effect of sex: *p* = 6.47705e–05, F(1,145) = 16.93) (Fig. 1i). Higher accuracy and increased correct choice repetition in female mice both contributed to females completing more rule shifts than males. These findings suggest that female mice may differ from males in the computations used in choice selection and reward learning.

### Stronger effects of choice bias and learning rate in female mice

Several broad computational mechanisms might simultaneously contribute to the sex differences we observe in cognitive flexibility. Differential reward updating [16, 40, 41], action repetition [15], and outcome sensitivity [17, 41] all influence sex differences in decision making. Our analyses suggest that female mice repeat correct choices more frequently, pointing to value updating and/or action repetition as likely computations driving sex-biased Set Shift behavior. To evaluate these potential mechanisms, we turned to an innovative combination reinforcement learning drift diffusion model (RLDDM) (Fig. 2a). With both reinforcement learning and perceptual decision making components, the RLDDM is well suited to model Set Shift behavior [4].

The RLDDM (Fig. 2a) was fit to female and male Set Shift behavior, allowing us to evaluate how strongly each model parameter (boundary separation, drift rate, bias, non-decision time, learning rate, forgetfulness, and surprise) is affected by sex. Boundary separation reflects the amount of evidence required to make a choice. Drift rate captures how quickly the model is driven toward a correct choice (evidence accumulation). The bias term represents the ability to pre-commit to the higher value choice based on the learned value differences between choices. Non-decision time captures the speed of sensorimotor processing–the time it takes to process the stimulus and execute a motor (choice) response. Learning rate (value updating) and forgetfulness (decay of the unchosen option) were included as part of the reinforcement learning component of the model. Overall, the RLDDM accurately modeled mouse Set Shift behavior (Supplementary Fig. 6).

Two main parameters associated with value updating and choice repetition were the strongest contributors to the sex differences observed in Set Shift behavior. As a primary effect, bias (pre-commitment to the higher value choice) was stronger in female mice compared to males (probability of direction (pd) = 99.05%, median = 0.9791, and 1.35% in region of practical equivalence (ROPE)) (Fig. 2b). This bias effect indicates that once the current rule choice is learned, female mice more strongly commit to that learned rule response. Learning rate was the next largest effect, with a stronger effect in female mice compared to males (pd = 92.20%, median = 0.5849, and 7.125% in ROPE) (Fig. 2b). This learning rate effect is consistent with prior literature [15, 40] and indicates a sex-bias in value updating. Together, these data suggest that computationally, female mice demonstrate increased cognitive flexibility during Set Shift by a combination of increased updating from learned values and repetition of higher value (rule) choices.

### Faster transitions out of exploration following rule shifts in females

Increased set shifting in female mice implicates increased cognitive flexibility compared to males. Cognitive flexibility can be formulated as the rate of adaptive transitions between exploration and exploitation. The optimal strategy for the task is to rapidly transition to exploration following a loss on a previous rule, and transition to exploiting a new rule as quickly as possible. We hypothesized that female mice showed increased cognitive flexibility because they were faster at at least one of these processes. Given that the RLDDM indicated higher learning rate in females during Set Shift, we further hypothesized that there should be no difference in the ability to become flexible (exploit→explore), but the ability to transition away from exploratory flexibility into a new rule state (explore→exploit) should be enhanced in females.

To identify latent explore-exploit states underlying Set Shift behavior, an input-output hidden Markov model (ioHMM) structure (Fig. 3a) was used, providing both behavioral information about the choices animals were making (left/right), as well as task information about the location of the light (left/right) to the model. With this input, the model estimates the most probable states that an animal is in on each trial. Our ioHMM models two types of cognitive states—exploration across rules, and exploitation of a certain rule (left/right/light). Compared to other models, ioHMMs have previously been shown to more accurately describe both neural and behavioral Set Shift data [19].

Male mice overall spent more time in exploration during Set Shift compared to females (two-sample *t* test: *p* = 0.0126, effect size = –0.0666) (Fig. 3b). To test the hypothesis that female mice specifically show increased transitions from exploration to exploitation, we quantified the percent of explore-labeled trials for the first five trials following rule shifts. Female mice, but not males, significantly decreased their exploration from the first to the fifth trial (two-sample *t* test: *p* = 0.0432, effect size = –2.9487), while males did not change their exploration over these trials and were significantly different from females (two-way repeated measures ANOVA effect of sex: *p* = 2.19611e–06, F(1,145) = 24.33) as their respective strategies further diverged across trials (Fig. 3c). This suggests that the ability to transition away from exploration in fewer trials largely contributes to the increased number of completed rule shifts observed in female mice. Indeed, analyzing the state dynamic landscape [16, 42] reveals the explore basin is shallower in females compared to males, allowing for easier transitions out of exploration (Supplementary Fig. 7a–c). However, transitioning away from exploration might mean transitioning towards exploitation of an incorrect rule state. To get a more comprehensive picture of how latent cognitive states change as the environment changes, we quantified the percent of trials in each of three states–explore, exploit (previous rule), and exploit (current rule)–five trials before and 10 trials following rule shifts. All animals similarly switch away from exploiting the previous rule (exploit previous rule state occupancy GLMM main effect of sex: *p* = 0.4285, β1 = –1.306; main effect of trial: *p* = 1.1011e–26, β2 = –2.028) and enter exploration when the rule shifts. Not only do female mice then exit exploration sooner than males (explore state occupancy GLMM main effect of sex: *p* = 0.0035, β1 = 7.6856; main effect of trial: *p* = 0.1690, β2 = 0.1195), they transition towards exploitation of the current rule within the first 10 trials following a rule shift while males do not (exploit current rule state occupancy GLMM main effect of sex: p = 0.0003, β1 = −2.709; main effect of trial: *p* = 1.5114e–82, β2 = 1.6112) (Fig. 3d, e). This effect of sex on exploitation following rule shifts persists regardless of the current rule type (side/light) (Supplementary Fig. 7h–k).

### Simultaneous exploitation of multiple rule states in females

In the Set Shift task, transitions away from exploration imply transitions towards exploitation of specific rules. However, it would take multiple trials to test all possible rule states, because each trial contains two response options, as well as two dimensions (side and light) to disambiguate. The rapid transitions into exploitation for females may not allow for adequate trials to fully disambiguate the rule. One possibility is that females may exploit multiple rules simultaneously.

**Fig. 4:**
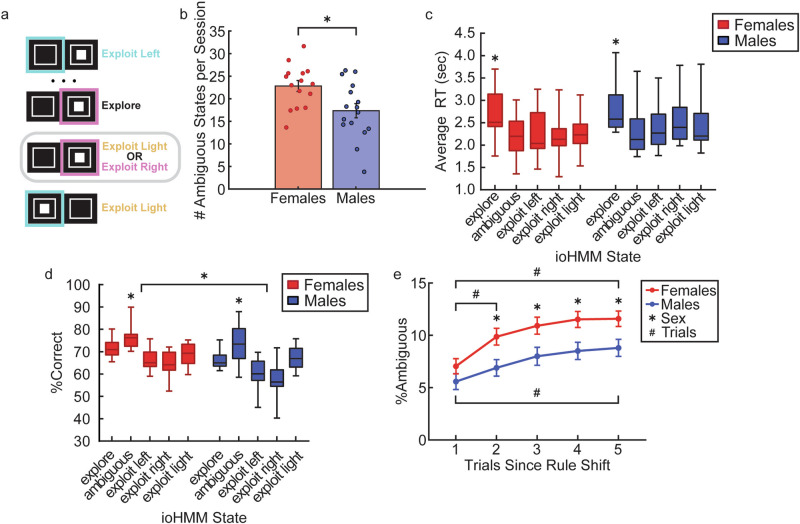
Simultaneous exploitation of multiple rule states in females. **a** Example schematic of an ambiguous state (outlined in light gray) occurring at trial t following an explore labeled state (t-1), where the animal’s choice and the location of the light remained unchanged from trials t-1 to trial t. The ioHMM gave an equal probability of two exploit states of different dimensions–at trial t, the animal could have been in the exploit light or exploit right state. At trial t + 1, the light stimulus moves and the animal chooses the side where the light is present, resulting in the ioHMM labeling trial t + 1 as an exploit light trial. **b** On average, female mice have significantly more ambiguous state trials (trials with two equiprobable states (an exploit side state and an exploit light state) compared to males (two-sample *t* test: p = 0.0105, effect size = 5.4744). **c** For female and male mice, response times (RT) were significantly slower during explore labeled states (GLMM main effect of explore state: *p* = 0.0009, β5 = 0.4605), compared to exploit labeled states and ambiguous states, which were similar in RT to exploit states. **d** For female and male mice, accuracy (average percent correct) was higher for ambiguous state trials compared to explore and exploit labeled trials (two-sample *t* test, comparison of female %correct in ambiguous and explore states: *p* = 0.0053, effect size = –5.4161; two-sample *t* test, comparison of male %correct in ambiguous and explore states: *p* = 0.0055, effect size = –6.8476). Overall, percent correct was higher in female mice regardless of state occupancy (two-way repeated measures ANOVA effect of sex: *p* = 6.46393e–07, F(1,145) = 27.11). **e** In all animals, the average percent of ambiguous trials significantly increased across trials following rule shifts (two-way repeated measures ANOVA effect of trial since rule shift: *p* = 5.77367E–06, F(4,145) = 8.163). In female mice only, the percent of ambiguous trials increased from 1 to 2 trials following a rule shift (two-sample *t* test: *p* = 0.0138, effect size = 2.831) and were overall higher on average compared to males (two-way repeated measures ANOVA effect of sex: *p* = 4.85193e–07, F(1,145) = 27.77). Figure 4b, e depict mean and SEM, Fig. 4c, d depict median (solid line) as well as min to max (whiskers).

With the external rule identity hidden from both the animal and the ioHMM, our exploit states are not mutually exclusive, allowing us to observe whether or not they are occurring in equal probability. On 10.39% of trials, the ioHMM gave an equal probability of the animal’s behavior being consistent with either of two states. These “ambiguous states” always occurred between the exploit light state and an exploit side state (Fig. 4a), with no significant differences between the percentage of ambiguous state labels assigned in each rule (two-way repeated measures ANOVA effect of rule: *p* = 0.1106, F(2,87) = 2.259) (Supplementary Fig. 8a).

Ambiguous states occurred more frequently for female mice (avg. 22.85 trials/session) than male mice (avg. 17.35 trials/session) (two-sample *t* test: *p* = 0.0105, effect size = 5.4744) (Fig. 4b), with no significant effect of training order (two-way repeated measures ANOVA effect of sex: *p* = 0.0229, F(1,27) = 5.822; effect of training order (last rule learned): *p* = 0.5155, F(1,27) = 0.4342) (Supplementary Fig. 8b). While ambiguous states may represent an “in-between” state where an animal is not exploiting a single rule, they are not explore states, as estimated by the model. This is supported by assessing average RT and accuracy in each state. Given the relationship between cognitive states, decision making confidence, and choice accuracy, previous research has identified that exploratory choices are slower and less accurate than exploitative choices [16]. Consistent with previous findings, RTs in explore-labeled trials are significantly slower than RTs in exploit states, regardless of sex (GLMM main effect of explore state: *p* = 0.0009, β5 = 0.4605) (Fig. 4c). RTs in ambiguous state trials did not significantly differ from RTs in other exploit states, providing evidence that ambiguous states are a type of exploit state. Overall, accuracy was significantly influenced by both sex and state (two-way repeated measures ANOVA effect of sex: *p* = 6.46393e–07, F(1,145) = 27.11; effect of state: *p* = 8.11006e–17, F(4,145) = 27.14). As an exploit state, ambiguous trials showed significantly higher accuracy compared to explore trials in both females (two-sample *t* test: *p* = 0.0053, effect size = –5.4161) and males (two-sample *t* test: *p* = 0.0055, effect size = –6.8476) (Fig. 4d). Accuracy was higher during ambiguous state trials compared to trials in all types of exploit states (left/right/light). This argues that ambiguous exploit state trials are unique, but the increased success of ambiguous state trials is unsurprising. Ambiguous states allow for commitment to two possible rules without needing to know the *specific* rule. These states occur during trials where the potential side rule and light rule are congruent, which are “easier” than incongruent trials where the light location is in conflict with the current side choice. As such, increased accuracy during ambiguous state trials is explainable by the statistics of the task.

Our analysis of ambiguous states suggests that these states represent trials where animal behavior co-occupies two unique exploit states. This is further supported by the relationship between trial-by-trial RLDDM value differences and the probability of ambiguous states from the ioHMM. Ambiguous states are mostly likely to occur when the RLDDM value estimate for both the light choice and one side choice are at their highest values (Supplementary Fig. 8c), meaning evidence is strong for choosing the light and a specific side simultaneously. To assess whether female mice transition to exploitation in fewer trials than males following rule shifts by simultaneously exploiting two states, we quantified the percent of ambiguous labeled states in the five trials following rule shifts–similar to our analysis of exploration following rule shifts. While the percent of explore labeled trials decreases in females shortly after rule shifts, the percent of ambiguous state trials increases after rule shifts in all animals (two-way repeated measures ANOVA effect of trial since rule shift: *p* = 5.77367E–06, F(4,145) = 8.163), with an overall higher occurrence in females (two-way repeated measures ANOVA effect of sex: *p* = 4.85193e–07, F(1,145) = 27.77) (Fig. 4e). Additionally, animals are most likely to occupy exploit states when exiting ambiguous states, indicating their commitment to a given rule choice (Supplementary Fig. 8d). This suggests that co-occupancy of two exploit states reflects a behavioral strategy of increased choice commitment to navigate periods of relative uncertainty during the Set Shift task and importantly, that female mice use this strategy more frequently.

In an effort to validate our ioHMM results, we analyzed and compared shuffled trial-by-trial state data (Supplementary Fig. 9). This method unpaired state labels from their assigned trials while retaining the overall number of trials in each state. Shuffling the order that states occurred in eliminated the influence of state-specific differences in RT and accuracy (Supplementary Fig. 9a,b). As expected, the shuffling did not change overall sex differences in these measures, but did eliminate temporal effects of rule shifts on state labels (Supplementary Fig. 9c,d). This further validates that the state assignments and measures we observe are not random effects, but rather reflect latent cognitive patterns/states.

Our ioHMM findings indicate that sex differences in Set Shift performance are largely influenced by differences in decision making strategy. When deliberating between rules during periods of uncertainty, female mice transition to exploitation more quickly than males by simultaneously exploiting two rule states.

### Task design impacts mouse Set Shift performance and disturbs previously observed sex differences

The initial version of the Set Shift task allowed 10 s after stimulus display for the animals to make a choice. If the animal did not make a choice within 10 s, the trial would timeout (logged as an omission), no reward would be received, and the next trial could be initiated following a 3 s ITI. After collecting 10 days of testing on the 10 s response limit Set Shift task, we tested two versions of the task where response time limits dropped to 3 s, similar to rat versions of the task [4] (Supplementary Fig. 10). The 3 s response time limit task reduced successful rule shifts in all mice, and temporarily reduced differences in performance across sexes. Moving animals back to a 10 s response time limit immediately improved performance (Supplementary Fig. 10c), suggesting that shortened deliberation times were deleterious to the cognitive strategies all animals used in the task, despite animals making decisions faster than 3 s in the original task (compare Fig. 1f). We also implemented a version where response time limits shortened from 10s to 3s with each successful rule shift. Despite the slower transition to shorter response time limits, performance at the 3 s limit was not improved compared to the base 3 s schedule, although there was a trend towards the re-emergence of the sex difference (Supplementary Fig. 10e–g). Additionally, there were no significant sex differences in the percent of omitted trials across task versions (Supplementary Fig. 11), all animals omitted significantly more trials when response time limits were shorter (Supplementary Fig. 11g). These data indicate that having adequate time to deliberate is a critical contributor to the sex differences we see in Set Shift behavior, consistent with our modeling results above.

## Discussion

Individual differences in the computations supporting cognitive flexibility may reveal mechanisms of neuropsychiatric risk and resilience. One critical variable influencing neuropsychiatric risk is sex. To directly assess sex-biased individual differences in cognitive flexibility, we developed a novel touchscreen Set Shift task for mice modifying successful designs for rats [4, 10]. Female mice demonstrated enhanced cognitive flexibility by completing more rule shifts with fewer errors, supported by enhanced commitment to exploiting rule choices sooner–sometimes because they commit to multiple rules simultaneously. This suggests that increased choice stability in female rodents enhances commitment to a strategy during periods of uncertainty and directly contributes to more rapid rule acquisition/performance. Our data supports recent conceptualizations of stability and flexibility as independent cognitive mechanisms, rather than opposite settings of the same mechanism [7], by demonstrating the somewhat surprising conclusion that a tendency towards exploiting options in decision making can drive enhanced cognitive flexibility in an operant Set Shift task.

Our data point to enhanced choice stability and reduced exploration in females compared to males as latent variables influencing sex differences in decision making across tasks. We have previously identified faster learning rates in female mice using a spatial restless bandit task [16], as well as stronger side choice biases in female mice using a visual restless bandit task [15]. Here, using a reinforcement learning drift diffusion model (RLDDM) [4], we similarly find stronger effects of learning rate and bias/pre-commitment in female mice. Using a hidden Markov model to understand explore-exploit bias in the restless bandit task, we found that female mice learn faster during exploration, transitioning to exploit states sooner than males who are overall more exploratory [16]. Here, using a modified input-output HMM, we again find that female mice were faster to transition into exploitation compared to males. Our findings are consistent with a broader literature showing sex-biased outcome sensitivity and learning rate during decision making. Female mice demonstrate stronger sensitivity to negative reward outcomes and risk [17, 43]. These effects may be related to sex differences in the dopamine system that are partially regulated by estrogen signaling [40, 44], and/or nonendocrine sex differences in behavioral variability and dopamine regulation [13, 45, 46].

Female mice were faster to transition into exploit states following rule shifts. Intriguingly, these exploit states were more likely to be “ambiguous”–trials where the behavior of the animal was consistent with multiple rule states. A key question is whether these states reflect actual computational and neural engagement of multiple rules at once. Ambiguous state decisions are faster than explore states and similar to single rule exploit states, consistent with the idea that exploration is more cognitively demanding than exploit decisions [16, 20, 47]. Likewise, the accuracy of ambiguous exploit state choices are *higher* than any other state. Ambiguous states always included the light and one side, occurring when the Q values for both the light and that side were at their highest, suggesting that there was evidence to exploit both if the opportunity arose. Given their success, and the equal opportunity that all mice have to utilize ambiguous states, it remains unclear why females are more likely to occupy ambiguous states. One possibility may be increased neural representation of rule states that supports cognitive flexibility [48] in female mice compared to males. A similar ambiguous “transition-like” state has been observed in neural data from the medial prefrontal cortex in some, but not all, male rats during a rule shifting task [49], and the establishment of neural representations in frontal cortex is needed for exploiting a rule [19]. It is unknown whether there are sex differences in encoding and representing rules, and future work may benefit from determining if the neural representation of these states impacts cognitive flexibility [50].

Adapting an operant Set Shift task for mice that permits temporal sensitivity has long been a goal in the field [9] given the genetic tractability of mouse models, and that high confidence analysis of neural dynamics during cognition often requires within-session measures. Our approach allows mice to shift between rules repeatedly within a session while permitting the high throughput and controllability of operant testing. We note several interesting features of the current design, including evidence that animals may sometimes anticipate rule shifts, demonstrated by increased exploration on the first trial of a new rule, and evidence that animals more successfully shift to and maintain the light rule, showing an advantage towards applying an established rule that is distinct from the difficulty of learning the light rule schema during shaping. Future modifications of this design could incorporate multiple response options in the visual dimension to further test intradimensional versus extradimensional shifts [9, 19, 29, 51–56]. This will further improve our operant design, which provides a well defined trial structure, a repeatable within-subjects design, and multiple within-session rule shifts with no investigator intervention required. This approach is well suited for addressing neurophysiological questions, as well as asking about sources of individual differences via genetic, pharmacological, viral, and circuit manipulations to further our understanding of neuropsychiatric risk and resilience.

## Supplementary information

Supplemental Materials

## Data Availability

The dataset generated and analyzed within the current study, as well as the code used for computational modeling (RLDDM and ioHMM) are available at https://github.com/gleww022/mouse-setshift.git.
