# Supplementary material for: Sex-biased computations underlying differential set shift performance in mice
Source: Neuropsychopharmacology. 2026 Apr 10;51(9):1669–79. doi: 10.1038/s41386-026-02397-z (PMC13389363; doi:10.1038/s41386-026-02397-z)
Supplement: Supplementary file 1 — Supplemental Materials [file 41386_2026_2397_MOESM1_ESM.docx]

# Supplemental Methods

## **Animals**

Thirty-two BL6129SF1/J mice (16M, 16F) were obtained from Jackson Laboratories (stock #101043). Animal counts were predetermined by a power analysis to detect the standard effect size of sex differences common in previous publications from the lab [15,16], targeting 80% power (G*Power 3.1). Animals were pre-exposed to the reinforcer (50% water diluted vanilla Ensure) in the home cage prior to operant training. Starting at 12 weeks of age, behavioral training and testing in the operant chambers occurred five days per week (Monday-Friday). Behavioral training and testing occurred in the same chamber for each animal and operant chambers were located in unique female and male behavior rooms to avoid potential confounds. Animals were fed each day following behavior.

##

## **Apparatus**

Sixteen triangular touchscreen operant chambers (Bussey-Saksida design, Lafayette Instrument Co., Lafayette, IN), enclosed inside sound attenuating cabinets were used for behavioral training and testing. Two of the three chamber walls were black, acrylic plastic. The third wall housed the touchscreen and was positioned directly opposite of the magazine. A two-hole mask over the touchscreen defined two possible choice options. The magazine provided Ensure liquid reinforcer (280 ms pump duration, ~7ul).

##

## **Pre-training**

Animals completed the Punish Incorrect schedule but the stimuli displayed on the touchscreen during the task were adapted to be consistent with stimuli presented during the Set Shift task. After mice reached criterion on each schedule (two consecutive days with 30 trials completed in 30 minutes or two consecutive days with 60 trials completed in 60 minutes, depending on schedule), they moved on to the next schedule. On average, animals spent five days on each schedule.

## **Rule Shaping**

Animals spent five days on each Rule Shaping schedule. Rule shaping schedules were 200 trials or 60 minutes long, whichever occurred first. Consistent with pre-training, animals needed to complete at least 60 trials (one per minute) for two consecutive days. An additional performance criterion was added to ensure that all animals could repeatedly select the correct rule choice, a key element of the full Set Shift task. Animals needed to also make at least 10 consecutive correct choices for two consecutive days before moving to the next schedule. This performance criterion was selected to ensure that all animals could repeatedly select the correct choice for longer than is necessary in the full Set Shift task, which required five consecutive correct choices to shift rules. While all animals performed more than 10 consecutive correct choices in each rule, we did not want to strictly require more than this limit to prevent overshaping or exclude specific decision making strategies (e.g. more exploratory animals which are more likely to be males). Each animal reached the shaping performance criterion of completing at least 10 consecutive correct trials, confirming that all animals learned each individual rule. Following exposure to each rule individually, testing began on the full Set Shift task.

## **Touchscreen Set Shift Task for mice**

Animals self-initate trials via nose-poke at the magazine when the tray light turns on. Following initiation, animals have a response time limit (10-3s depending on schedule) during which they must execute a choice before a 3s timeout begins. Incorrect choices similarly evoke this timeout, paired with an incorrect tone (3000 Hz). After each choice or omission, a 3s inter-trial interval (ITI) exists before animals can initiate the next trial. Immediately following a correct choice, the magazine distributes liquid reward (Ensure) and simultaneously the tray light turns on. Once reward is collected, the tray light turns off and the ITI starts.

## **Set Shift schedule modifications to test reduced response time limits in mice**

The baseline Set Shift task allowed a 10s response time limit. To assess the ability of mice to perform the task with the same response time limit as rats in a standard operant chamber [[4,10]](https://paperpile.com/c/uC29o8/UptR8+2lT9m), a second Set Shift schedule was created with a response time limit of 3s. An additional descending response time limit schedule was developed to further investigate how deliberation times influenced behavior during Set Shift. This schedule started with a 10s response time limit that decreased by 1s after each completed rule shift until the response time limit reached 3s where it remained for the duration of the session. Given that the response time limit decreased with each completed rule shift, not all animals reached 3s in each session. One female and two males only reached 3s during some sessions of this schedule. In those cases, the number of rule shifts completed during the 3s response time limit was recorded as zero. One male animal never reached the 3s response time limit and as such, could not contribute data to the analysis of this schedule.

## **Reinforcement Learning Drift Diffusion Model (RLDDM)**

We used a reinforcement learning drift diffusion model (RLDDM) to model decision making in the Set Shift task [4]. This model was run on data from sessions 5-10 of Set Shift to avoid initial learning effects. This model combines elements of two prominent computational modeling approaches for decision making: 1) a reinforcement learning model, which updates trial-to-trial value learning, and 2) a drift diffusion model, which models evidence accumulation up to choice. While RL models can accommodate side biases, they often struggle on their own to deal with shifting dimensions of decision making (e.g., RL models usually only accommodate a single dimension of choice (side or image/light)). It has been previously demonstrated that an RLDDM (Reimer, Dastin van Rijn, et al. 2024) accommodates the features of this task that are perceptual as well as those that are reinforcement driven. The RLDDM is capable of accounting for numerous possible task inputs that traditional RL models are not always able to (accuracy history, light location, and side options) as well as possible task outputs (choice and response time).

Here, the RLDDM was fit to mouse Set Shift behavior (the sequences of choices and response times each session) using Markov-Chain Monte Carlo with the HDDM Python package [4,33]. The hierarchical levels of the Bayesian fitting approach are group (female/male) to individual. Most parameters (learning rate, boundary separation, drift rate, non decision time, forgetfulness, bias, and surprise) have their own group level intercept which is the hierarchical root for all animals of the corresponding sex. Initial values for the side and light features, baseline bias, and non decision time variability are estimated at the population level (one parameter value for the entire dataset). To ensure reliable estimation of parameters, four independent chains (2,000 total samples) were run for each model and convergence was confirmed by assessing whether the Gelman-Rubin statistic was <1.1 [4,34]. We next evaluated how strongly each model parameter (boundary separation, drift rate, bias, non-decision time, learning rate, forgetfulness, and surprise) is affected by sex. Boundary separation reflects the amount of evidence required to make a choice. Drift rate captures how quickly the model is driven toward a correct choice (evidence accumulation). The bias term represents the ability to pre-commit to the higher value choice based on the learned value differences between choices. Non-decision time captures the speed of sensorimotor processing–the time it takes to process the stimulus and execute a motor (choice) response. Learning rate (value updating) and forgetfulness (decay of the unchosen option) were included as part of the reinforcement learning component of the model.

Using the RLDDM, values are assigned to each choice based on estimates of the current side (left/right) and “light” values. The model uses those values to generate a choice using a drift diffusion process, accumulating evidence until the decision boundary is reached. Trials with a larger total difference in value between choices are more likely to have shorter response times and more consistent choices. Depending on whether a choice is rewarded, values are updated using a reinforcement learning process (**Figure 2a**). Modeling the data from each group (females/males), the posterior distribution of group model parameter differences inform which computations show the largest sex biases. The full set of equations for the RLDDM:

The value of a choice (V) is the sum of its respective side and light values (Q):

$V(side,t) = Q(side,t) + (Light(t) == side) \times Q(light,t)$

Drift ($v(t)$) on each trial is directed proportionally to the side with the highest value:

$v(t) = v_{0} \times(V(Left,t)-V(Right,t))$

Bias ($\beta(t)$) on each trial is directed proportionally to the side with the highest value, ignoring the light, where $\phi$ is the logistic equation exp(x)/(1+exp(x)) which is used to ensure that bias is kept between 0 and 1. The baseline bias factor is estimated at the population level and accounts for any non-learning related bias to one side of the chamber or the other. Scaling bias accounts for bias based on learned association between a side and rewards. Scaling bias is measured separately for females and males.

$\beta(t)= \phi(\beta_{0}+ \beta_{1}\times(Q(Left,t)- Q(Right,t))$

On each trial, non-decision time ($\tau(t)$) was sampled from a uniform distribution with mean $\tau_{0}$ and width $\tau_{var}$:

$\tau(t) \sim Uniform(\tau_{0},\tau_{var})$

For each trial, response times (RT) follow a Wiener First Passage Time (WFPT) distribution with parameters for drift ($v(t)$), boundary separation ($\alpha$), bias ($\beta(t)$), and non-decision time ($\tau(t)$):

$RT(t) \sim WFPT(v(t),\alpha,\beta(t),\tau(t))$

Separate non-linear learning rates ($\delta$) were calculated for each chosen feature ($C$) to model surprise ($\gamma$):

$\delta(C,t)= \delta_{0}|r(t)- Q(C)|^{\gamma}$

The value of each chosen feature was updated via a standard Rescorla-Wagner learning rule:

$Q(C,t+ 1)= \delta(C,t)\times(r(t)- Q(C,t))$

The value of unchosen features diminishes according to a forgetfulness factor ($F$):

$Q(C,t+ 1)= Q(C,t)\times(1-F)$

The RLDDM has 11 parameters in total: initial values for the side and light features, drift rate $(v_{0})$, baseline bias ($\beta_{0}$), scaling bias ($\beta_{1}$), non-decision time ($\tau_{0}$), variability of non-decision time ($\tau_{var}$), boundary separation ($\alpha$), baseline learning rate ($\delta_{0}$), surprise ($\gamma$), and forgetfulness ($F$). Parameters were estimated using a Bayesian hierarchical approach. To determine the effect of sex on model parameters, the group-level distributions for the parameters were assessed using the probability of direction (PD) and the region of practical equivalence (ROPE) [4,35]. PD refers to the proportion of the parameter distribution greater than 0 with a value above 0.5 (indicating a general increase in the parameter across groups) and a value below 0.5 (indicating a general decrease in the parameter across groups). ROPE refers to the proportion of the parameter distribution that falls within a region that is practically equivalent to a null-effect (+/- 0.1). PD indicates the existence and direction of an effect while ROPE establishes significance.

## Input-Output hidden Markov model (ioHMM)

An input-output hidden Markov model (ioHMM) was used to label latent cognitive states underlying Set Shift behavior. The HMM framework assumes that choices are generated from some unobserved latent cognitive states. Our model identified two general types of cognitive states–exploration and exploitation–defined by their unique patterns of choice. Given that omitted trials do not contain choices, the ioHMM cannot model omissions. Here, choice behaviors are modeled as emissions (observations) from one of the four distinct latent cognitive states–explore, exploit left, exploit right, and exploit light (**Figure 3a**). In the explore state, the emission probability for specific choice types are uniform, meaning that the generation of all choice types (left/right/light) are equally probable. The exploit states only emit the type of choice being exploited (e.g. the exploit left state only generates left choices). The transition matrix fit to the subject captures the unique transition probability between these four states in each individual. To disambiguate between choice dimensions (side/light), the location of the light cue was used as an input layer into the ioHMM. Together, the final ioHMM receives each animals’ choice sequences and the respective locations of the light cue, and outputs the most probable latent state for each trial.

The base ioHMM MATLAB code was adapted from Ebitz et al., 2019 and Ebitz et al., 2020 [19,20], and Kevin Murphy’s MATLAB HMM toolbox. A detailed description of the ioHMM:

Within the HMM framework, choices or “emissions” (y) are generated by an unobserved decision process within some hidden, latent state (z). These latent states are defined by both the probability of making each choice (k) out of (N_k_) possible options given the state of the system (e.g. whether the light is on the left or right side) and the probability of transitioning from each state to every other state. This framework was extended to allow inputs, such as the location of the light, to influence the probability of observing each emission in each state [36]. During exploration, animals have an equal, but random probability of making left and right choices regardless of the light location.

$p(y_{t} = k|z_{t} = explore) = \frac{1}{N_{k}}$

However, the location of the light is critical for disambiguating between the different rule states. For example, if an animal is following the left rule, they will repeatedly make left choices regardless of what side the light is on. If an animal is exploiting the light rule, they will only make left choices when the light is on the left side and right choices when the light is on the right side. The observation model for rule states was dependent on whether or not the observation (choice + light location) met the condition for the current rule:

$p(y_{t} = k|z_{t} = rule_{i},k \in rule_{i}) = 1$

$p(y_{t} = k|z_{t} = rule_{i},k \notin rule_{i}) = 0$

The latent states in this model are Markovian, meaning that they depend only on the most recent state (z_t_) and the most recent location of the light (l_t_), and are independent of time:

$P(z_{t}|z_{t-1},l_{t-1},y_{t-1}, . . . ,z_{1},l_{1},y_{1}) = P(z_{t}|z_{t-1},l_{t-1})$

The probabilities of each state transition were described by the one-time-step probability of transitioning between every combination of past and future states (i,j). Unlike the emissions matrix, the transition matrix was not influenced by the location of the light.

$p(z_{t} = i|z_{t-1} = j)$

The model had four possible states (one explore state and three rule (exploit) states). Parameters were tied across exploit states such that each exploit state had the same probability of beginning from exploration and of sustaining itself. Transitions from exploration to the exploitative states were similarly tied, making it equally likely to start exploiting any of the three rule states after exploration. This ensures that for each individual, the relationship between transition probabilities remains the same, but the actual values of those transition probabilities differ as the matrix is fit to each individual. Rule sequences were pseudo-randomized to prevent prediction of the next rule, including the starting rule. Transitions directly between exploit states were not permitted and choices were initialized in the explore state. The model assumed that mice had to pass through the explore state in order to gain enough information to start exploiting a new rule, even if only for a single trial. This modeling choice reduced the number of free parameters to estimate, allowing us to estimate fewer parameters more accurately with a limited number of trials. As such, the model had two free parameters.

The model was fit via expectation-maximization using the Baum Welch algorithm [37]. This algorithm identifies a (possibly local) maxima of the complete-data likelihood based on the joint probability of the latent state sequence and the sequence of observed choices. The algorithm was reinitialized with random seeds 20 times, and the model that maximized the observed (incomplete) data log likelihood was selected as the best for each session. To label latent states from choices, the Viterbi algorithm was used to identify the most probable *a posteriori* sequence of latent states [38,39].

The ioHMM is fit in part by identifying a set of equations that describes the rate at which individuals explore, exploit, and transition between states. Laurie et al., 2024 describes analytical methods used to illustrate the landscape of state dynamics, namely their stationary distributions and activation energies [42]. These landscapes or state basins visualize the energy needed to exit and transition between states–shallower basins indicate that less energy is required to exit that state whereas deeper basins require more energy to exit. State basin analysis was conducted on data from all Set Shift sessions (all sessions 1-10) (**Supp. Figure 7b**), as well as just the second half of sessions (end sessions 5-10) (**Supp. Figure 7c**) when behavioral stability was reached to avoid potential confounds from animals changing strategies across learning.

# Supplemental Results

## Intra- and extra-dimensional shifts

We find that regardless of sex, accuracy (average percent correct) is higher during EDS compared to IDS. This is evident whether we observe across all 10 sessions of Set Shift or looking just at Set Shift sessions 5-10, when behavior stabilizes (**Supplemental Figure 5**). We further analyzed errors in the trials following rule shifts for IDS and EDS in female and male mice. We did not find any sex differences so we are also showing data for all mice combined. Unsurprisingly given the above plots, errors in EDS are lower sooner following rule shifts.

One key difference that may be contributing to behavioral differences between IDS and EDS is that while the side dimension has two rules (left and right), there is only one kind of rule in the light dimension (select the “light” cue). While this is a limitation that we hope to address in future versions of this task, numerous tasks based on a similar design share this limitation but have still revealed important neural findings (Darrah et al., 2008; Reimer, Dastin van Rijn, et al., 2024). Unlike non-operant Set Shifting tasks, one type of shift is not overtrained before testing the other in the touchscreen operant Set Shift task. Additionally, many operant rat Set Shifting tasks focus solely on extra-dimensional shifts only, where the light rule alternates between side rules. However, our operant Set Shift task allows for a wider range of shift types.

## Ambiguous states

The reasons why female mice are performing trials that are more likely to be labeled by our ioHMM as continuing multiple exploit states (ambiguous) could include differences in motivational parameters, including the value updating or learning rate as assessed by our RLDDM and consistent with prior literature of enhanced RL value updating in females (Chen et al., 2021; Orsini et al., 2022; Golden et al., 2023). However, we do not believe that these effects are due to greater female responding to light cues, for several reasons. First, the RLDDM strongest sex difference identified was a “bias” term, which represents increased precommitment to a particular side according to the q value of the sides. This suggests that part of what may enhance set shifting in females is a reduced tendency to explore sides during learning, consistent with a sex difference we have previously seen in mice in a visual bandit task (Chen et al., 2021). In **Supplemental Figure 7**, we instead identify that both males and females have increased exploit state duration for light rules compared to side rules, suggesting that both sexes have a “respond to light” bias, but that therefore this is unable to explain the sex difference in ambiguous state occupancy. Collectively, we conclude that increased side commitment in females, paired with a bias towards responding to the light shared by all animals in the task, leads to greater ambiguous state occupancy in females. Indeed, in **Supplemental Figure 8c**, we demonstrate that ambiguous exploit states are most likely to occur in animals when Q values are high for the light and for one side much more than the other, suggesting that from an RL perspective the evidence for choosing that side and the light are equally strong. If males have reduced value updating rates as indicated, then they may be less likely to reach high Q values for a side and the light simultaneously.

It is possible that during ambiguous states, the true latent state of the animal is the exploitation of a single rule, but that the ioHMM simply cannot discern which one. However, we do not have enough evidence to support that animals are *only* exploiting a single rule during ambiguous states. Value estimates are difficult to dissociate in congruent trials; therefore, it is difficult to argue that animals are only learning about one rule when they are getting evidence about two rules. If values are updating for two congruent rules, animals might not be strategically exploiting two rules, but that is what we are able to pick up from their behavior. For example, given that ambiguous states occur when the light is on the correct side and has not moved, one might argue that ambiguous states are simply exploit light states during which the light has not moved. If that were the case, we would expect that when exiting the ambiguous state (e.g. the trial is no longer congruent), animals would overwhelmingly occupy the exploit light state; however, we do not see this. What we do find is that animals are most likely to occupy the exploit states and least likely to occupy the explore state (**Supplemental Figure 8d**). While animals are more likely to occupy the exploit light state than the exploit side states following ambiguous states, this is expected given the statistics of the task–selection of the light cue during uncertainty is the optimal solution with the highest probability of reward. Ambiguous states might truly be ambiguous because animals do not default into one particular state once the ambiguity has passed. Ultimately, we believe the final resolution to this question requires neural data.

#

# **Supplemental Figures**


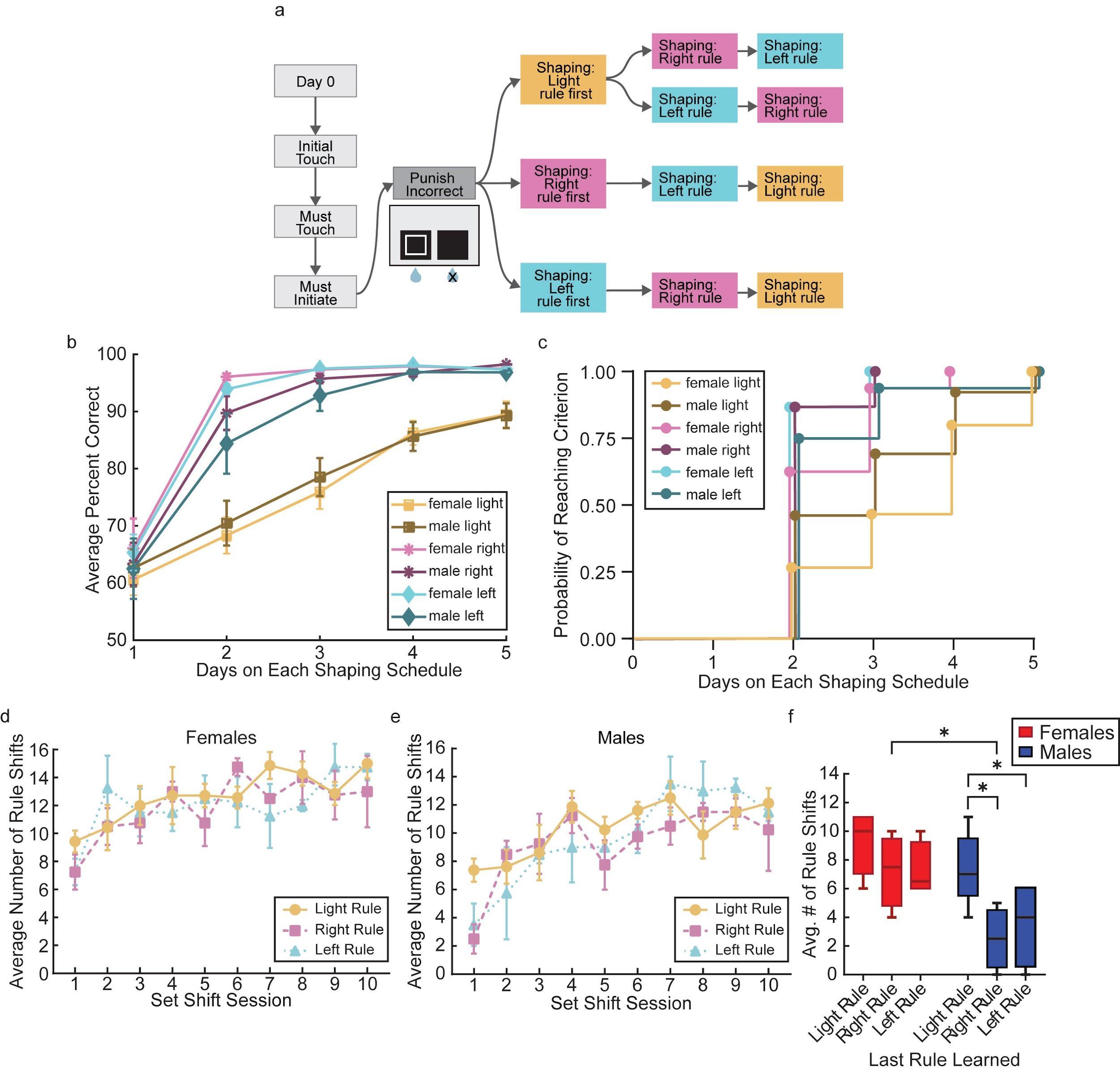


**Supp. Figure 1.** (a) Schematic of the training schedules that mice complete prior to testing on the Set Shift task. Schedules in light grey (Day 0, Initial Touch, Must Touch, and Must Initiate) have been previously described in Chen et al., 2021 and are used to train basic touchscreen use. For the Set Shift training pipeline, Punish Incorrect has been modified to reinforce that the touchscreen is active when the outlined white square is present on the screen. After Punish Incorrect, animals learned each rule individually and were counterbalanced across shaping schedules. (b) By the end of the fifth daily session of each shaping schedule, all animals, regardless of sex or training order, learned each rule as measured by average percent correct. By day five on each shaping schedule, sex differences in performance of each individual rule stabilized. After learning, performance on the Side Rule shaping schedules was significantly higher compared to Light Rule shaping on day five regardless which rule dimension animals learned first (two-way repeated measures ANOVA effect of shaping rule: p=9.79162e-10, F(2,86)=26.66; effect of sex: p=0.8917, F(1,86)=00186). (c) All animals completed five daily sessions of each shaping schedule to ensure adequate exposure to each individual rule. All animals met the rule shaping criteria (completion of at least 60 trials with 10 consecutive correct choices for two consecutive days) within five days on each shaping schedule. Criteria was met for most animals between session two and three for side rules and two and five for light rules. (d-e) Prior to the first daily session of the full Set Shift task (Set Shift Session 1), animals completed individual rule shaping where each rule was learned individually. Training order groups in this figure represent the rule shaping that animals completed last, immediately preceding testing on the full Set Shift task. (d) Average number of rule shifts that females in each training group (light shaping last (n=7), right shaping last (n=4), and left shaping last (n=4)) completed throughout the 10 sessions of the Set Shift task. (e) Average number of rule shifts that males in each training group (light shaping last (n=8), right shaping last (n=4), and left shaping last (n=4)) completed throughout the 10 days on the Set Shift task. (f) Overall, the average number of rule shifts completed during Set Shift Session 1 was influenced by the last shaping rule that animals completed and sex (two-way repeated measures ANOVA effect of training order (last rule): p=0.0022, F(2,25)=7.883; effect of sex: p=0.0004, F(1,25)=16.50). Directly comparing performance of each group (sex by training order) revealed that between groups, female mice that completed right shaping last completed significantly more rule shifts on the first day of Set Shift compared to males that completed right shaping last (two-sample t-test: p=0.0276, effect size=-4.75). Within sex effects were also found–males that completed side shaping last performed significantly fewer rule shifts during Set Shift Session 1 compared to males that completed light shaping last (two-sample t-test left-light comparison: p=0.0323, effect size=-3.875; right-light comparison: p=0.0055, effect size=-4.875). Supp. Figure 1b, 1d, and 1e depict mean and SEM, and 1f depicts median (solid line) as well as min to max (whiskers).

#

#


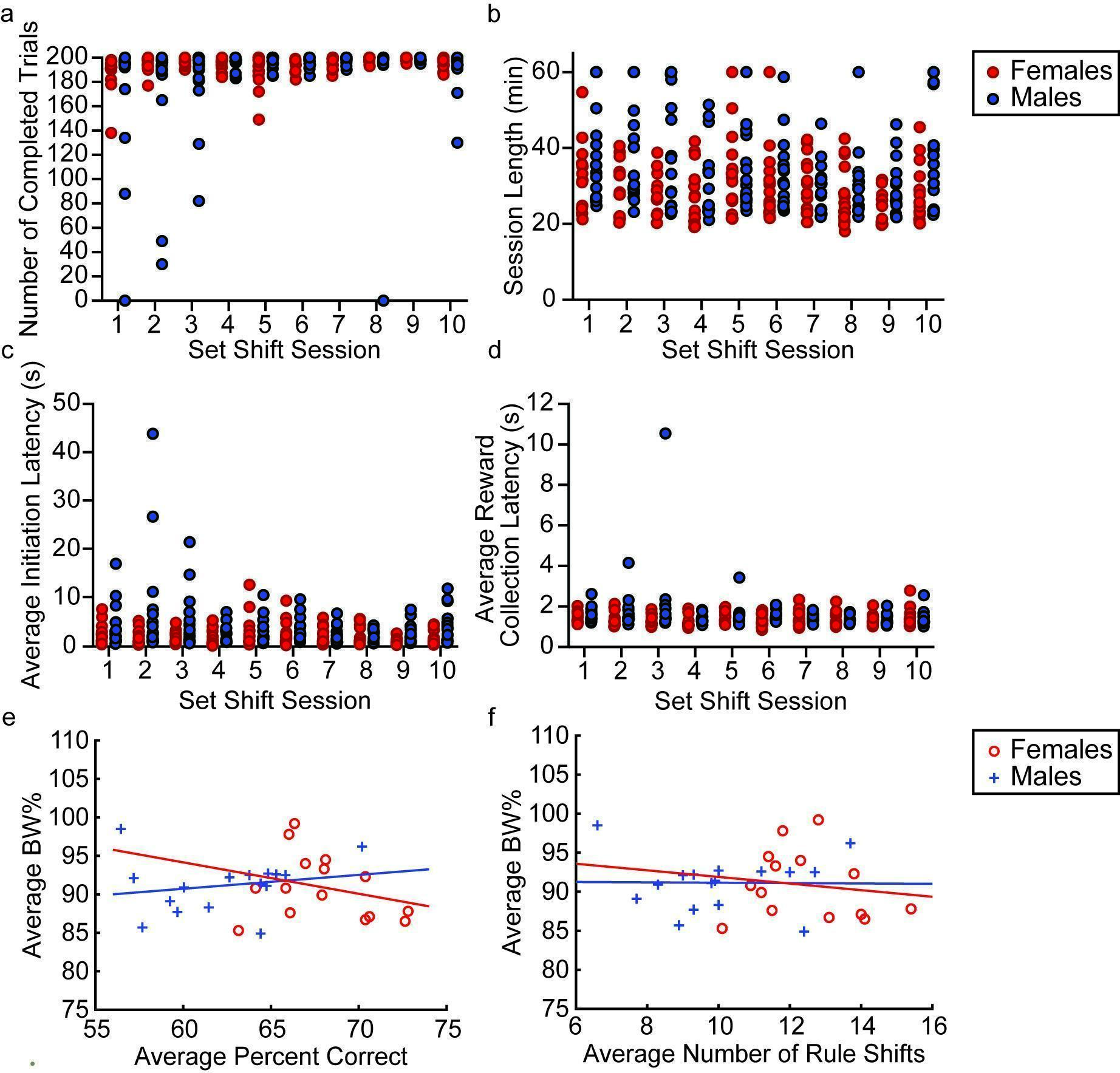


**Supp. Figure 2.** (a) Number of completed trials across daily Set Shift sessions. On average female mice completed 194.39 daily trials while male mice completed 188.56 daily trials. (b) Length in minutes of Set Shift sessions across days. Daily Set Shift sessions end after the completion of 200 trials or 60 minutes. On average, sessions were 28.95min long for females and 34.10min long for males. (c) Average latency (seconds) to initiate each trial across daily Set Shift sessions. Average initiation latency was 2.13s in female mice and 3.87s in male mice. (d) Average latency (seconds) to collect reward across daily Set Shift sessions. Average reward collection latency was 1.39s in female mice and 1.55s in male mice. (e) Average percent of baseline body weight (food restriction amount) was not significantly correlated with Set Shift performance (average percent correct) in females (correlation: r = -0.2791, p = 0.3138), nor in males (correlation: r = 0.1939, p = 0.4717). (f) Average percent of baseline body weight (food restriction amount) was not significantly correlated with the average number of completed rule shifts in females (correlation: r = -0.1488, p = 0.5967), nor in males (correlation: r = -0.0130, p = 0.9618). Supp. Figure 2a-d depicts individual averages and 2e-f depicts individual averages as well as the line of best fit for each group (females and males).


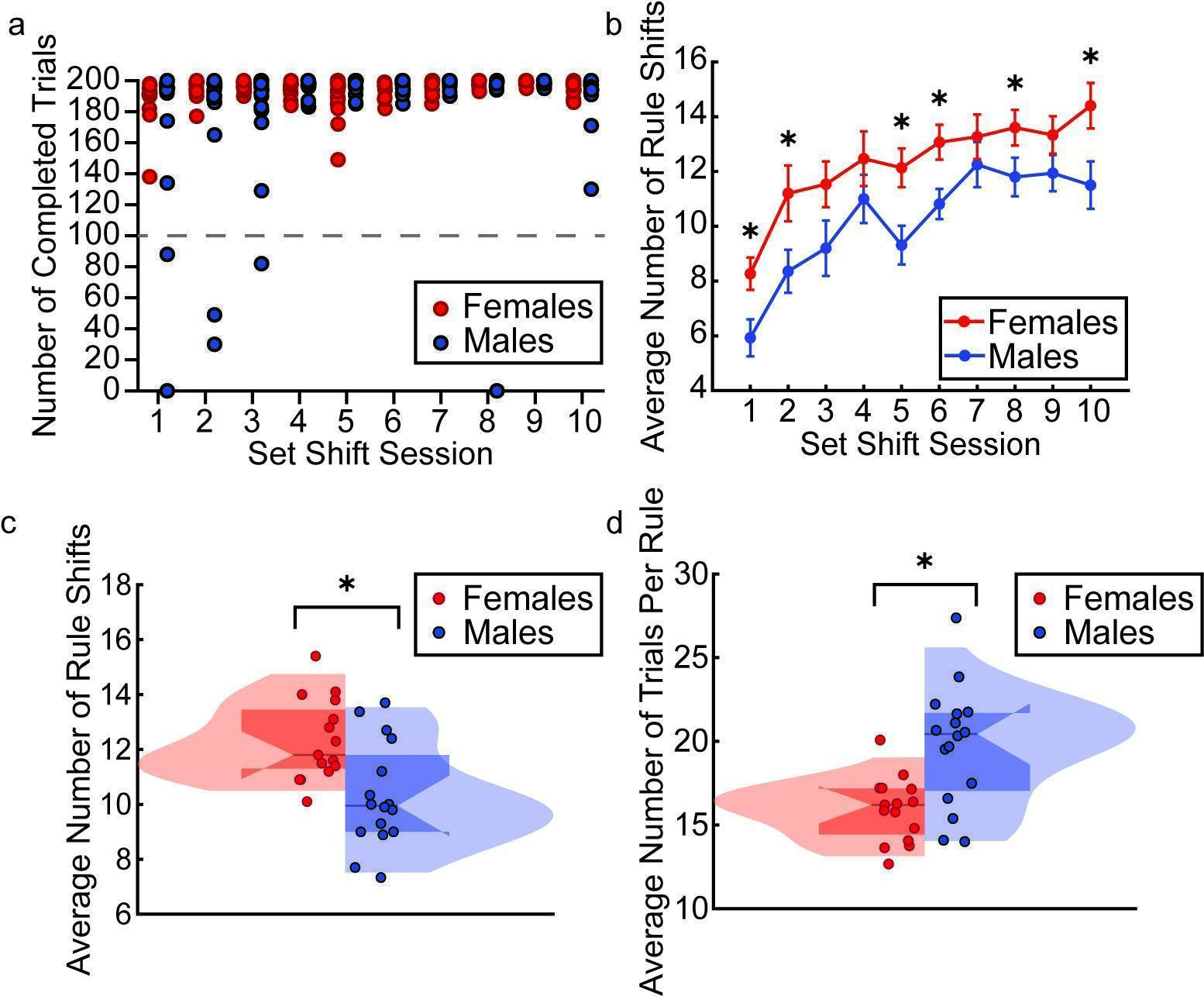


**Supp. Figure 3.** Re-analysis of main findings excluding session data from animals that completed fewer than 100 trials in a given session. (a) Visualization of the six cases where an animal completed fewer than 100 trials in a session (data points below black dotted line). (b) Excluding data from specific mice during sessions where they completed fewer than 100 trials does not change the original finding from Figure 1c. Though Set Shift performance (number of completed rule shifts) improves for all animals across the 10 total days on Set Shift, female mice consistently complete more average rule shifts compared to males across daily sessions (two-way repeated measures ANOVA effect of sex: p=6.51012E-09, F(1,284)=35.82

Effect of session: p=4.8604E-14, F(9,284)=10.51). (c) Excluding data from specific mice during sessions where they completed fewer than 100 trials does not change the original finding from Figure 1d. Averaging across Set Shift days, female mice complete significantly more rule shifts than males (two-sample t-test: p=0.0026, effect size=2.0373). (d) Excluding data from specific mice during sessions where they completed fewer than 100 trials does not change the original finding from Figure 1e. Female mice reach criterion (5 consecutive correct choices without omissions) in fewer trials than males on average, spending less time in each rule and rule shifting faster than males (two-sample t-test: p=9.5810e-04, effect size=-3.8272). Supp. Figure 3a depicts individual averages, 3b depicts mean and SEM, and 3c-3d depict median (solid line), 1-99th percentile of data in maximum shaded area vertically and kernel density horizontally, and inner hourglass shaded area depicts 25-75% confidence interval of data.


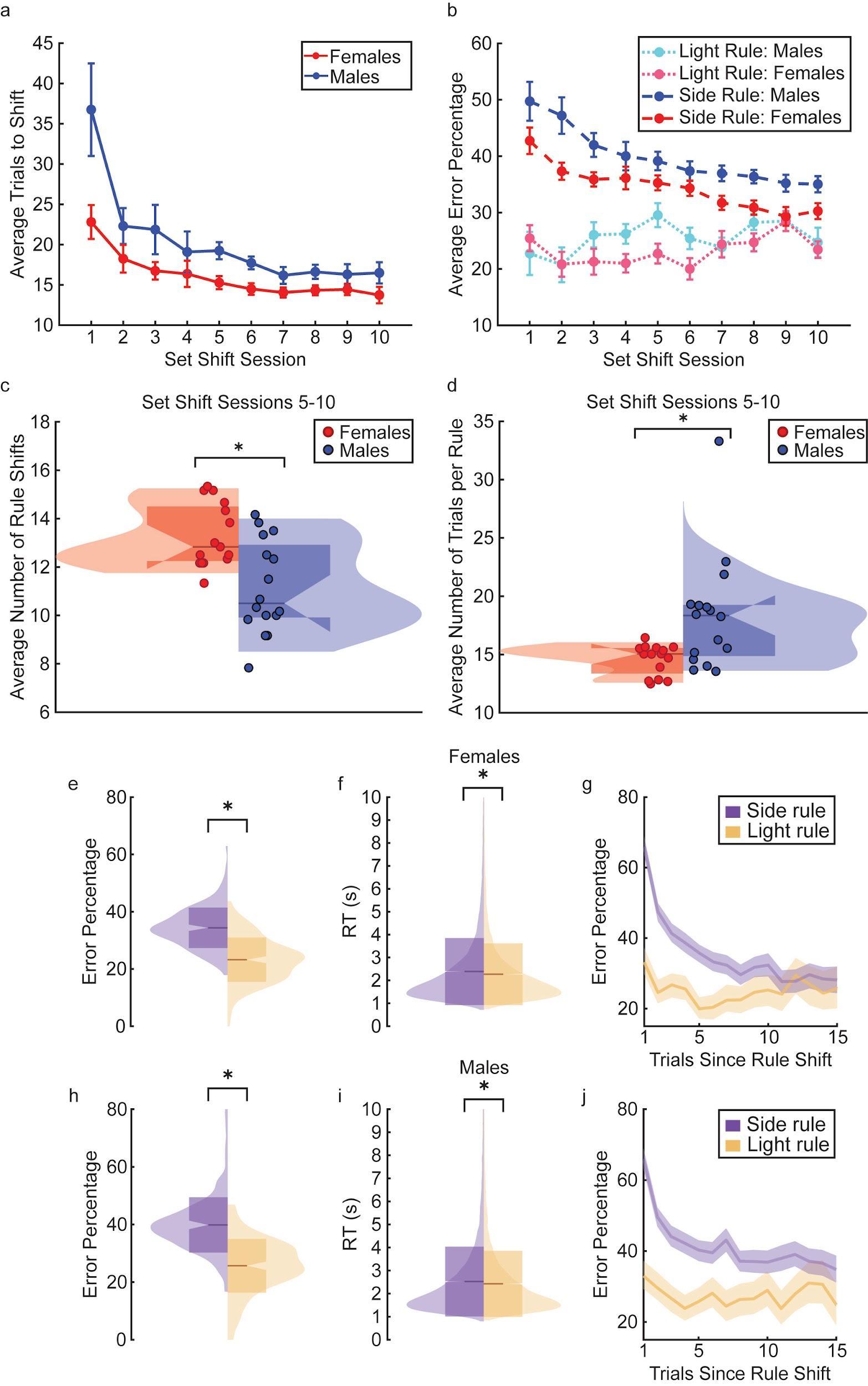


**Supp. Figure 4.** (a) Across sessions, all mice meet criterion to rule shift (five consecutive correct choices) in fewer trials, plateauing at session 5-6 for both female and male mice (two-way repeated measures ANOVA main effect of sex: p=2.133E-06, F(1,286)=23.42; main effect of session: p=1.5093E-13, F(9,286)=10.14). (b) Side rule performance improves (decrease in average error percentage) across Set Shift sessions. In contrast, animals consistently perform the light rule with fewer errors across Set Shift sessions regardless of sex however, female mice perform both side and light rules with fewer errors compared to males across Set Shift sessions (GLMM main effect of sex: p=4.9097e-05, β1=0.2281; main effect of session: p=0.0002, β2=-0.0439; main effect of rule (side vs. light): p=8.1574e-66, β3=-0.7381; session*rule interaction: p=4.5229e-17, β4=0.0563). (c-d) Examining only data from sessions 5-10 (after initial Set Shift task learning) does not change our main Figure 1 findings. (c) Female mice still perform significantly more rule shifts than males sessions 5-10 (p=0.0011, effect size=2.1542). (d) Similarly, females during sessions 5-10 still meet criterion to rule shift in fewer trials than males (p=0.0070, effect size=-3.7968). (e-g) Comparison of response times (RTs) and errors in each rule type (side and light) in female mice. (e) Female mice perform significantly fewer errors during light rules compared to side rules (GLMM main effect of rule: p=3.1655e-62, β1=-0.4618). (f) There is a significant difference in female RTs during side and light rules (GLMM main effect of rule: p=2.5352e-11, β1=0.1064). (g) Visualization of errors in each rule type (light versus side) 1-15 trials following rule shifts in female mice. (h-j) Comparison of RTs and errors in each rule type (side and light) in male mice. (h) Male mice perform significantly fewer errors during light rules compared to side rules (GLMM main effect of rule: p=1.6661e-59, β1=-0.4358). (i) There is a significant difference in male RTs by rule type (GLMM main effect of rule: p=8.4023e-17, β1=0.1421). (j) Visualization of errors in each rule type (light vs. side) 1-15 trials following rule shifts in males. Supp. Figure 4a-b, 4g, and 4j depict means and SEM. Supp. Figure 4c-f and 4h-i depict median (solid line), 1-99th percentile of data in maximum shaded area vertically and kernel density horizontally, and inner hourglass shaded area depicts 25-75% confidence interval of data.


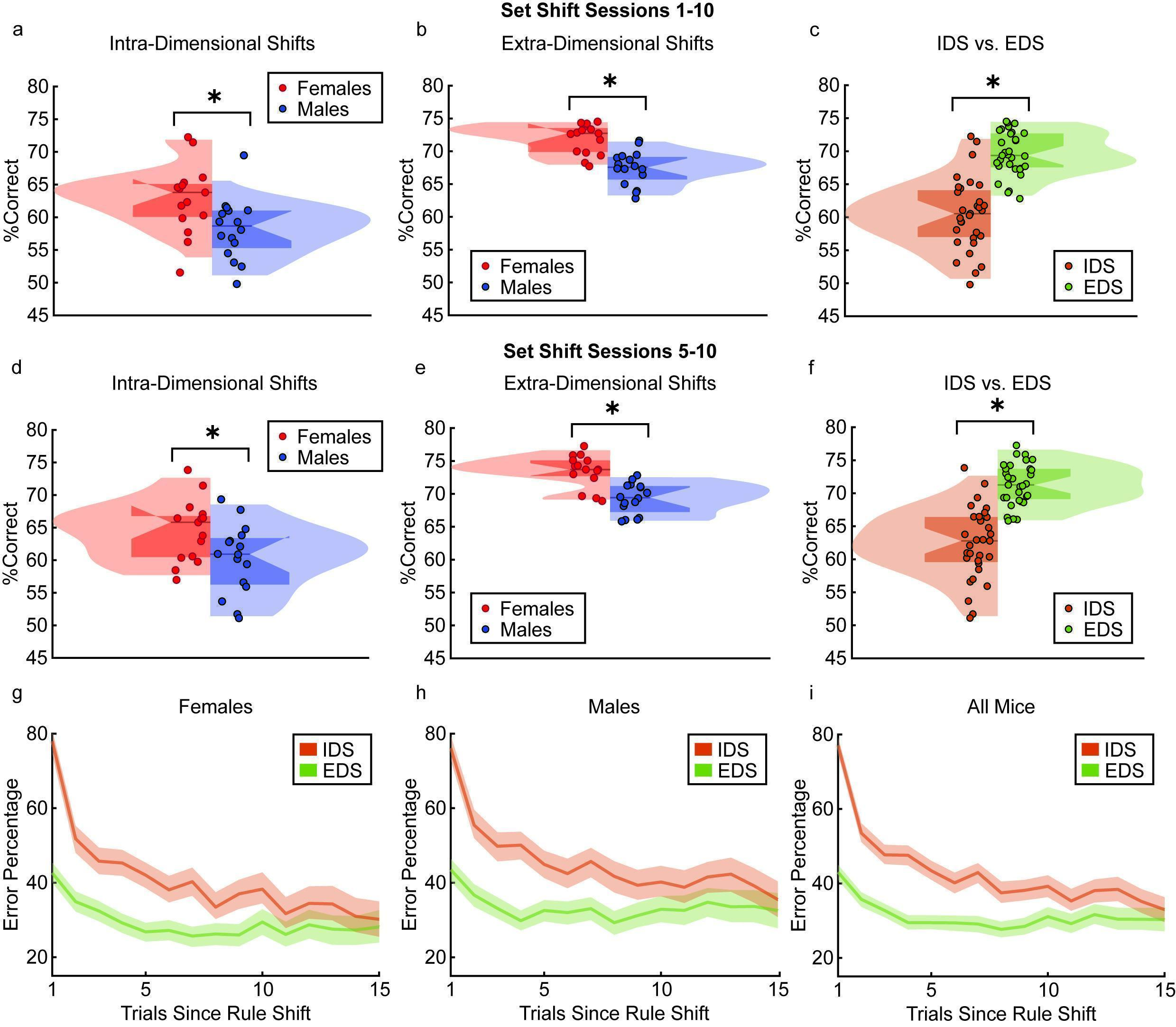


**Supp. Figure 5.** Analysis of intradimensional (side to side) and extradimensional (side to light or light to side) rule shifts. (a-c) Data from Set Shift sessions 1-10. (a) Female mice demonstrate higher accuracy during intradimensional shifts (IDS) compared to males (two-sample t-test: p=0.0219, effect size=4.5756). (b) Female mice are more accurate during extradimensional shifts (EDS) compared to males (two-sample t-test: p=1.5687e-05, effect size=4.6647). (c) All animals are more accurate (higher %Correct) during extradimensional shifts compared to intradimensional shifts (two-sample t-test: p=2.0689e-10, effect size=-9.0758). (d-f) Data from Set Shift sessions 5-10, after initial Set Shift task learning. (d) Female mice still demonstrate higher accuracy during IDS compared to males (two-sample t-test: p=0.0244, effect size=4.3154). (e) Female mice remain more accurate during EDS compared to males (two-sample t-test: p=3.9011e-05, effect size=4.1767). (f) Accuracy during EDS remains higher compared to IDS (two-sample t-test: p=6.2174e-11, effect size=-8.9732). (g-i) Visualization of errors in IDS vs. EDS 1-15 trials following rule shifts in females (g), males (h), and in all mice combined (i). Supp. Figure 5a-f depicts means and SEM. Supp. Figure 4c-f and 4h-i depict median (solid line), 1-99th percentile of data in maximum shaded area vertically and kernel density horizontally, and inner hourglass shaded area depicts 25-75% confidence interval of data. Supp. Figure 5g-i depicts means and SEM.


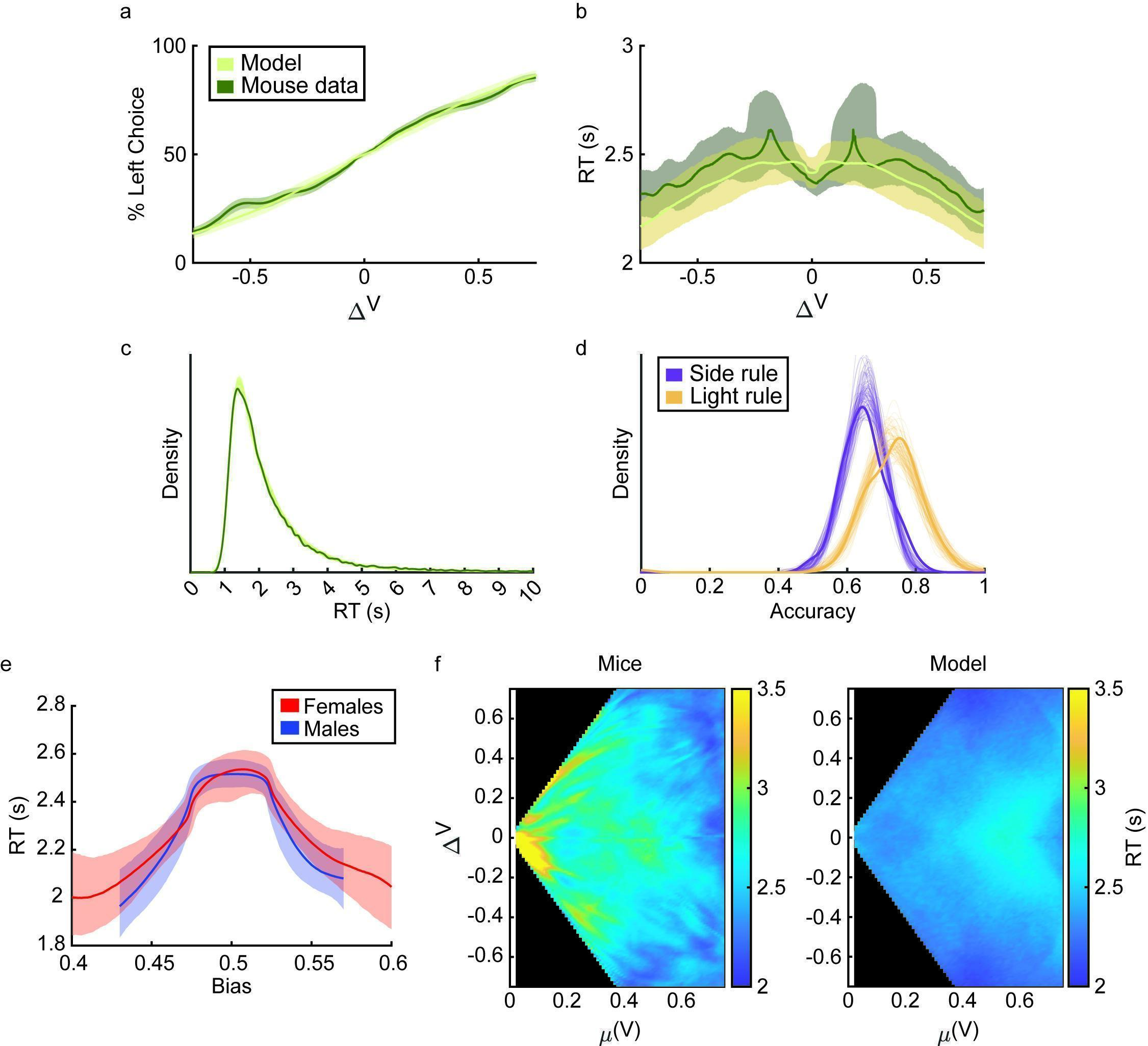


**Supp. Figure 6.** (a) This plot assesses how frequently left choices are made in the model and mouse data based on the differences between choice (left and right) values (𝚫V). In both the modeled data (light green) and mouse behavioral data (dark green), the left choice was selected least when its value was low and most when its value was high. (b) Response times (RTs) (seconds) were fastest when the value between each choice (𝚫V) differed the most, and slower as the values became more similar in both the mouse data (dark green) and model data (light green). At 0, the value of each choice is equivalent and RTs again quicken as selection of either choice produces the same outcome. (c) Overall, RTs from the model (light green) did not significantly differ from RTs in the mouse data (dark green). d) Mouse choice accuracy (transparent) in each rule type (side: purple, light: yellow) were similarly captured by the model data (opaque). (e) There are no sex differences in the overall relationship between bias and reaction times, although there is a strong contribution of bias to reaction times such that greater bias is associated with quicker choices. The RLDDM identifies that female mice have greater scaling bias, which means that the range of trial-to-trial bias values that they can reach is also greater. This explains why the male line (blue) ends before the female line (red) on either end of the plot. (f) Additional analysis to further understand the dip in mouse RTs at 0 in panel b that does not appear in the model data. Plotting the difference in total value between choices (y-axis) against the mean value of the available options (x-axis) for the mice and the model data reveals higher response times (RTs; yellow in the heatmap/colorbar) in the mouse data when the mean value of the available choices (μ(V)) is low (i.e., few recent rewards experienced) and when the difference in total value (ΔV) is also low. This phenomenon is not present in the model data. Mice may be slower to respond in these cases due to low motivation during periods with fewer rewards. Supp. Figure 6a, 6b, and 6e depict the 95% interval of the posterior simulations (shaded region).


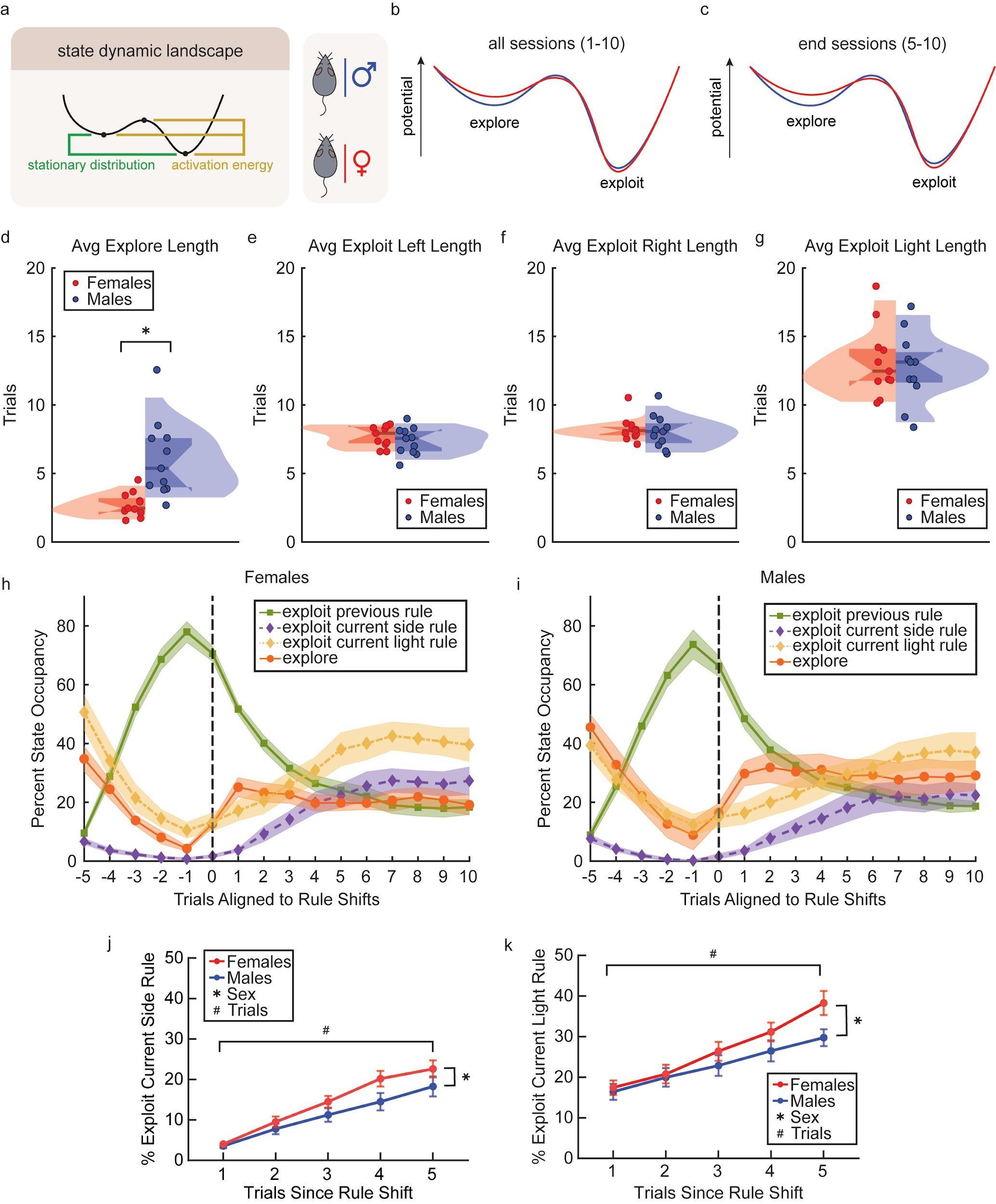


**Supp. Figure 7.** (a) The landscape of state dynamics or state basins visualize the energy needed to exit and transition between states–shallower basins indicate that less energy is required to exit that state whereas deeper basins require more energy to exit. Across all Set Shift sessions (all sessions 1-10) (b), as well as just the second half of sessions (end sessions 5-10) (c), the explore basin is shallower in females state data compared to males, allowing for easier transitions out of exploration. (d-g) Average dwell time in each state assessed by quantifying the length of each state bout (number of trials). (d) Average dwell time in the explore state is significantly longer in male mice compared to females (two-sample t-test: p=0.0013, effect size=-3.3655). (e) There are no significant sex differences in the number of trials spent in each exploit left bout (two-sample t-test: p=0.3329, effect size=0.3775), (f) exploit right bout (two-sample t-test: p=0.7181, effect size=0.1674), (g) nor exploit light bout (two-sample t-test: p=0.6754, effect size=0.4723). (h-i) Expansion of the analyses in Figure 3d-e. In these plots, the listing of trials is counting the number of trials that have passed since the rule shifted. Therefore, trial 0 (the trial where the rule shifts) means that no trials have happened prior to this one in the current rule. Similar to the original version of these plots, the solid green line is the percent of trials where mice are exploiting the previous rule (exploit previous rule) (the active rule on trials -5-0) and the orange line represents the percent of trials labeled explore (explore). Unique to these plots, the percent of trials where the mouse is exploiting the current rule (the rule that is active on trials 0-10) is split based on whether the current rule is a side (exploit current side rule) or the light rule (exploit current light rule). Regardless of sex, all animals more quickly transition to exploiting the current light rule compared to exploitation of the current side rule (two-way repeated measures ANOVA effect of rule comparing across all animals: p=4.1487E-143, F(1,960)=927.6). (j) Across trials 1-5 following rule shifts, female mice exploit the current side rule more than males (two-way repeated measures ANOVA effect of sex: p=0.0040, F(1,145)=8.537; effect of trial since rule shift: p=5.97328E-19, F(4,145)=31.66). (k) Across trials 1-5 following rule shifts, female mice exploit the current light rule more than males (two-way repeated measures ANOVA effect of sex: p=0.0129, F(1,145)=6.340; effect of trial since rule shift: p=2.69156E-11, F(4,145)=16.73). Supp. Figure 7d-g depicts median (solid line), 1-99th percentile of data in maximum shaded area vertically and kernel density horizontally, and inner hourglass shaded area depicts 25-75% confidence interval of data. Supp. Figure 7h-k depicts mean and SEM.


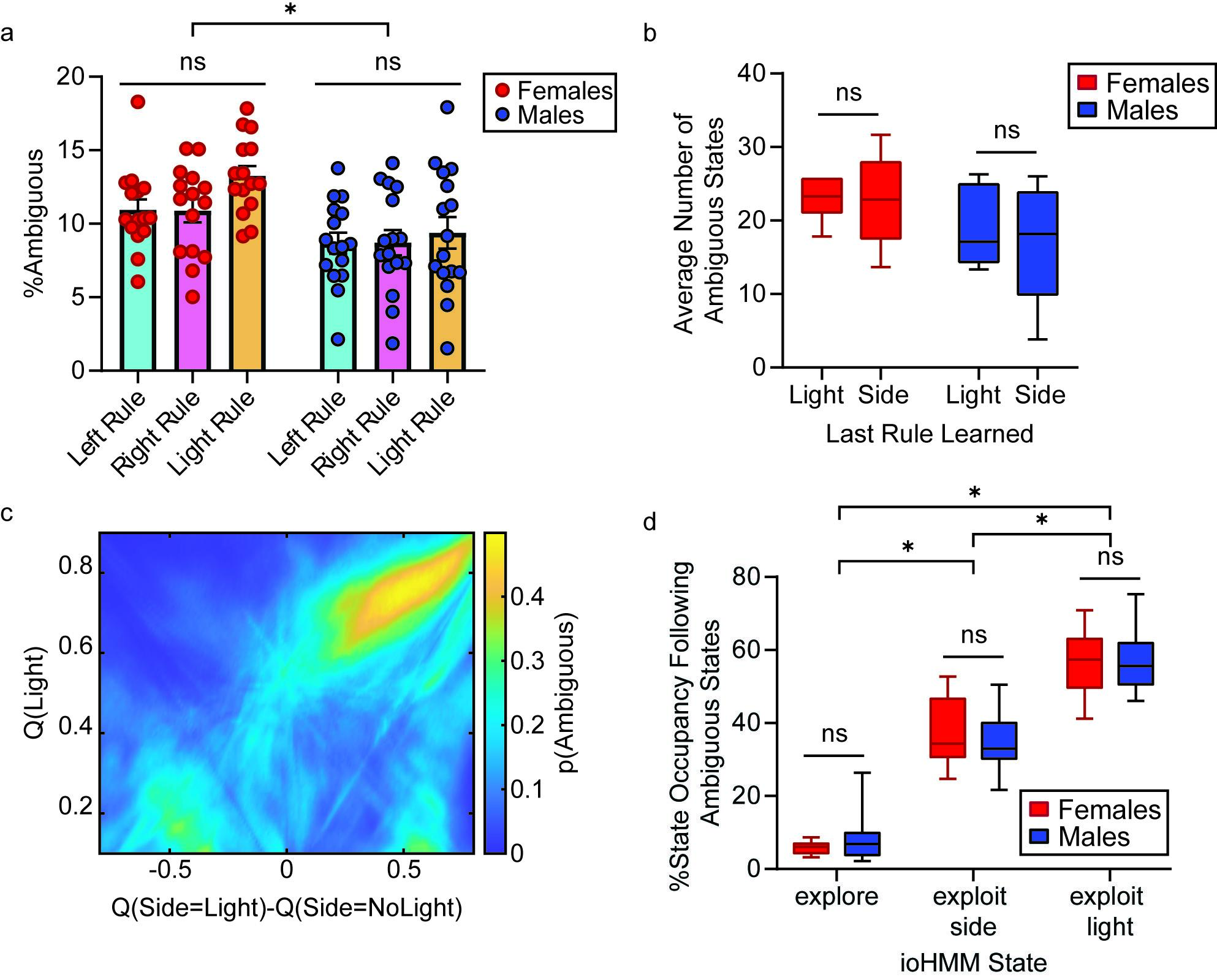


**Supp. Figure 8.** (a) There are no significant differences between the percentage of ambiguous state labels assigned in each rule (two-way repeated measures ANOVA effect of rule: p=0.1106, F(2,87)=2.259). Consistent with Figure 4b, the percent of ambiguous labeled states is higher in female data compared to males (effect of sex: p=8.44046E-05, F(1,87)=17.02). (b) There are no significant differences in the average number of ambiguous states by training order, however the significant effect of sex remains (two-way repeated measures ANOVA effect of sex: p=0.0229, F(1,27)=5.822; effect of training order (last rule learned): p=0.5155, F(1,27)=0.4342). (c) The highest probability of ambiguous states (yellow in the heatmap) is seen when the value estimate for both the light choice and one side choice are at their highest values. The probability of ambiguous state (p(Ambiguous)) is reflected in the heatmap/colorbar. The y-axis is the estimated value for the light. The x-axis represents the estimated value of the illuminated side (the side with the light cue present) minus the estimated value of the side without the light cue (e.g. the difference between the side with the light cue and the side without the light cue). Ambiguous states are most likely to occur when the estimated value for the light is high and the estimated value for the illuminated side is high. This is consistent with the hypothesis that the animal has gathered evidence for either a side rule or a light rule, with one side rule being eliminated based on its low value estimation. (d) Following ambiguous states, animals are most likely to occupy the exploit light or exploit side state and least likely to occupy the explore state, regardless of sex (two-way repeated measures ANOVA effect of ioHMM state: p=2.5906e-42, F(2,87)=349.6, effect of sex: p>0.9999, F(1,87)=1.557e-020). Occupancy of each state (explore, exploit side, and exploit light) significantly differed from each of the other states during the trial following ambiguous ioHMM state labels (paired t-test explore-exploit side comparison: p=1.69231e-15, effect=29.33, explore-exploit light comparison: p=7.78181e-23, effect=50.17, exploit side-exploit light comparison: p=9.18779e-08, effect=20.84). Supp. Figure 8a depicts mean and SEM, and 8b and 8d depict median (solid line) and min to max (whiskers).

# **
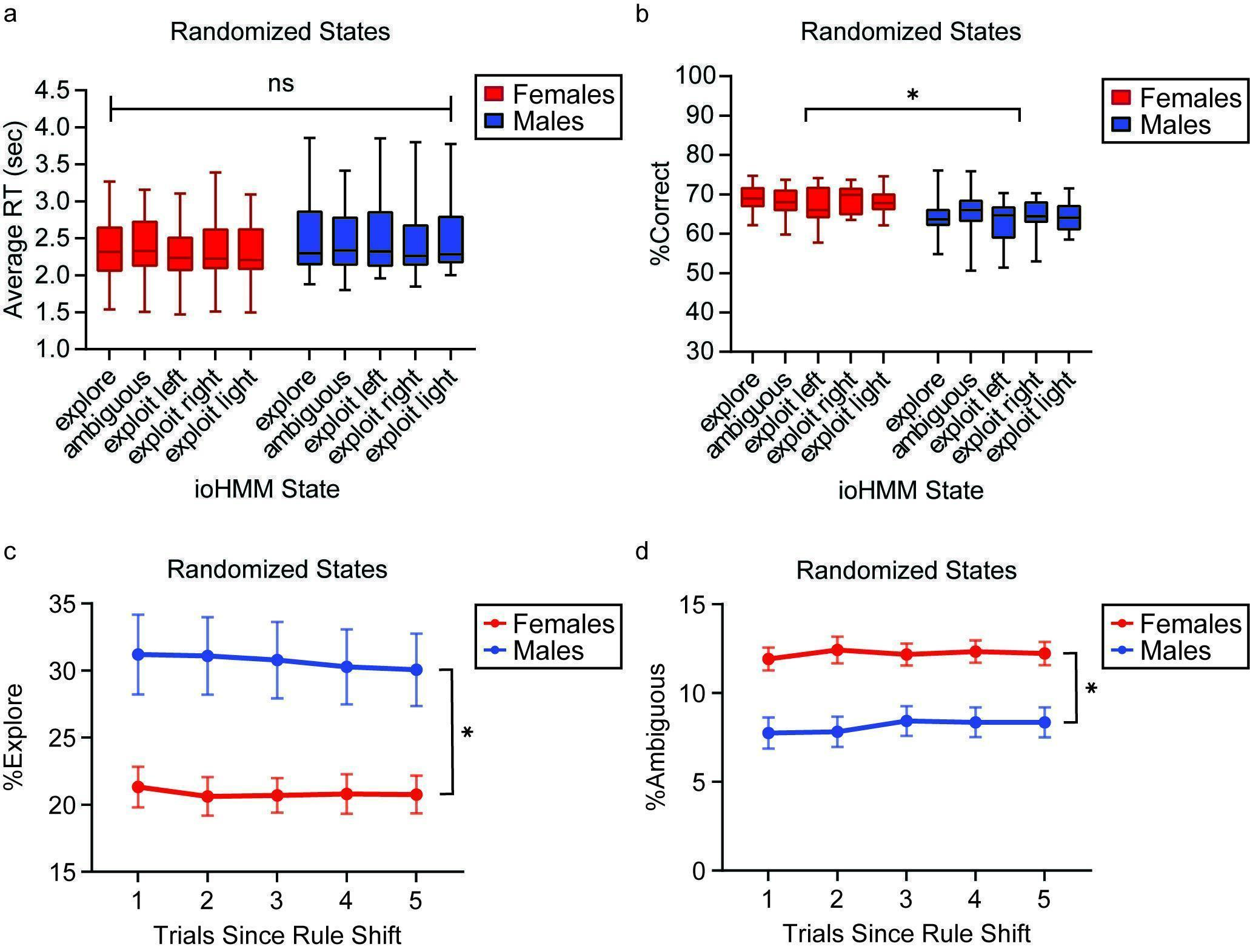
**

**Supp. Figure 9**. To validate our ioHMM findings, we randomized the trial-by-trial state sequences for each animal and each session of Set Shift. This allowed us to uncouple state labels from their respective trials without changing the overall composition of states for each animal. (a) No effect of state on average response time (RT) when state labels are randomized across trial sequences (two-way repeated measures ANOVA effect of state: p=0.9961, F(4,145)=0.0451; effect of sex: p=0.071, F(1,145)=3.307). (b) No effect of state on average percent of correct trials when state labels are randomized (two-way repeated measures ANOVA effect of state: p=0.5025, F(4,145)=0.839). The overall effect of sex on percent correct remains, with female mice demonstrating higher accuracy compared to males (effect of sex: p=3.30226e-07, F(1,145)=28.66). (c) After randomizing state labels, the effect of trial (temporal structure) is no longer significant on the percent of explore labeled trials 1-5 trials following rule shifts (two-way repeated measures ANOVA effect of trial since rule shift: p=0.9969, F(4,145)=0.0405). The overall effect of sex on exploration remains, with increased exploration in male mice (effect of sex: p=2.81896e-10, F(1,145)=45.97). (d) The effect of trial on percent of ambiguous labeled states (trials with two equiprobable exploit states) is no longer significant when state labels are randomized across trials (two-way repeated measures ANOVA effect of trial since rule shift: p=0.9636, F(4,145)=0.1481). Overall, the number of ambiguous states in each sex remains unchanged, with significantly more ambiguous states occurring in female mice (effect of sex: p=3.44438e-14, F(1,145)=70.80). Supp. Figure 9a-b depicts median (solid line) and min to max (whiskers), and 9c-d depicts mean and SEM.


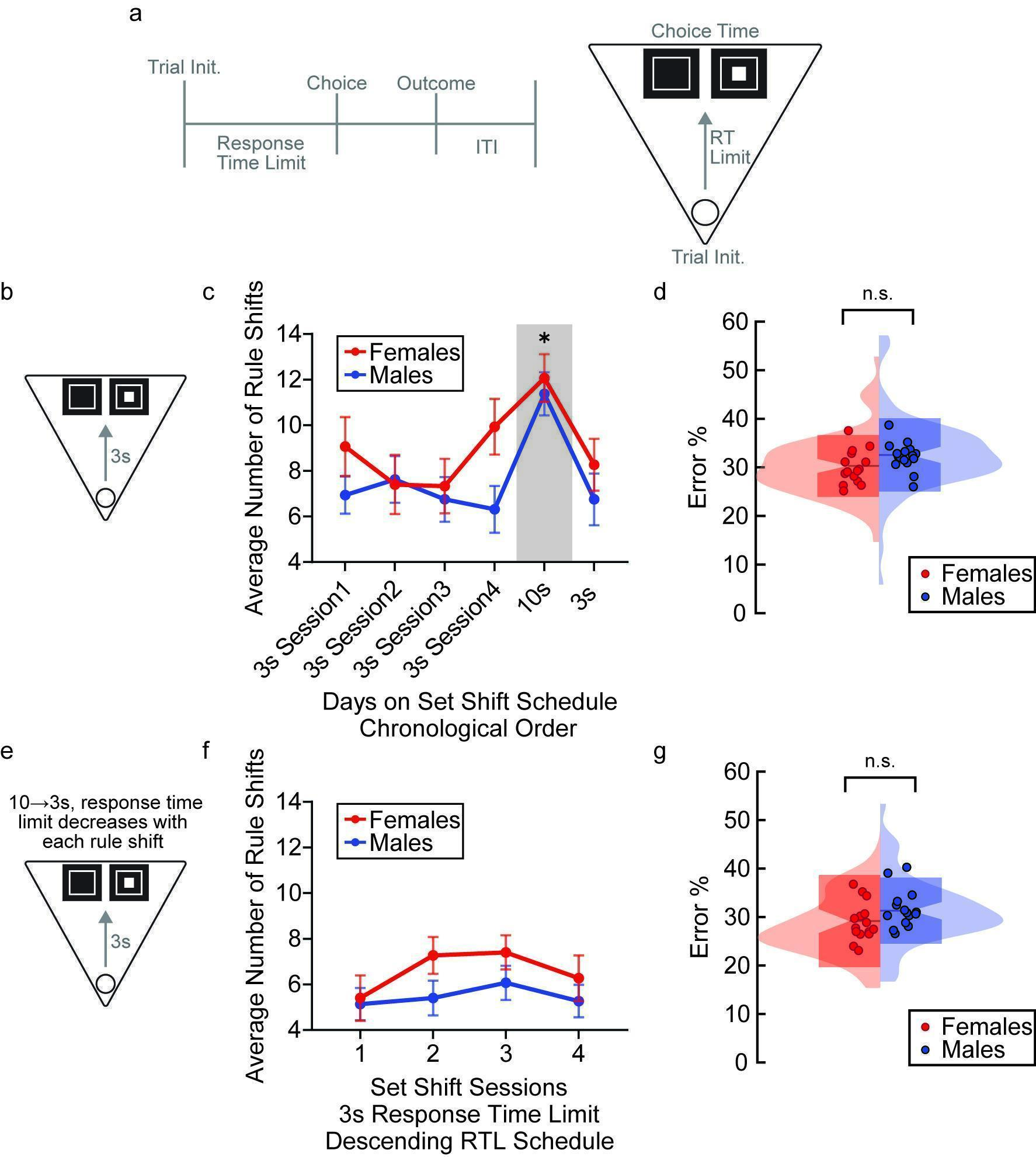


**Supp. Figure 10** (a) Schematic representation of the trial structure for all Set Shift schedules. After trial initiation, the animal has 10-3s to make a choice (response time limit) depending on schedule. The response time limit (RT Limit) is represented in the top-down chamber illustration by an arrow from trial initiation at the magazine to choice time at the touchscreen. (b) Illustration of the Set Shift schedule with a 3s response time limit. (c) The average number of rule shifts that female (red) and male (blue) mice completed during each chronological day on the 3s Set Shift schedule. After four days on the 3s schedule, animals were again tested on our standard Set Shift schedule which allowed a 10s response time limit. The average number of completed rule shifts significantly improved during the 10s Set Shift schedule (two-way repeated measures ANOVA effect of schedule: p=0.0003, F(5,174)=4.887) and again fell when tested on the 3s Set Shift schedule the following day (two-sample t-test comparison of average number of rule shifts during chronological Set Shift sessions 10s and 3s in females: p=0.0202, effect size=-3.8; and in males: p=0.0038, effect size=-4.625). Overall, testing on a schedule with a 3s response time limit minimized sex differences in the average number of rule shifts completed. (d) The effect of sex on the percent of errors performed during Set Shift is no longer significant during the 3s Set Shift schedule (GLMM main effect of sex: p=0.3955, β1=0.1195). (e) Illustration of the descending Set Shift schedule where the response time limit starts at 10s and decreases by 1s with each completed rule shift until the response time limit reaches 3s. Data from this schedule in f and g are specifically from trials at the 3s response time limit. (f) Trend towards significant effect of sex on the average number of rule shifts completed at the 3s response time limit in this schedule (two-way repeated measures ANOVA effect of sex: p=0.0561, F(1,112)=3.727). (g) No significant sex difference in the percent of errors during trials at the 3s response time limit within this schedule (GLMM main effect of sex: p=0.4964, β1=0.0882). Supp. Figure 10c and 10f depict mean and SEM. Supp. Figure 10d and 10g depict median (solid line), 1-99th percentile of data in maximum shaded area vertically and kernel density horizontally, and inner hourglass shaded area depicts 25-75% confidence interval of data.


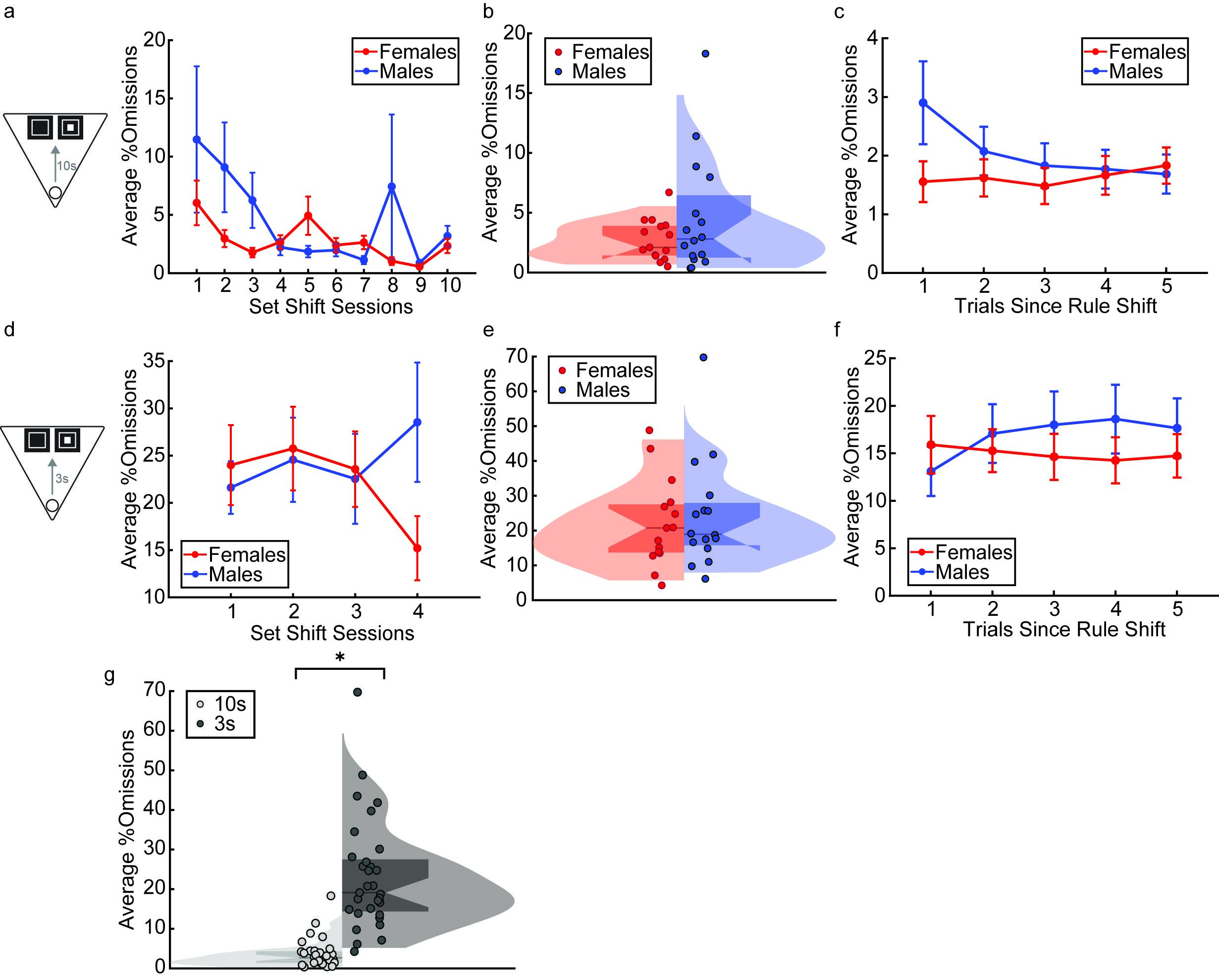


**Supp. Figure 11** If an animal does not make a choice within the response time limit during the Set Shift task, the trial is logged as an omission. (a) Animals completed consecutive 10 sessions of the Set Shift task with a 10 second response time limit. There was no significant effect of sex (two-way repeated measures ANOVA effect of sex: p=0.0920, F(1,290)=2.857) or of session (two-way repeated measures ANOVA effect of session: p=0.0566, F(9,290)=1.867) on the average percent of omitted trials (Average %Omissions). (b) Averaging across sessions of this task version revealed no significant effect of sex on the average percent of omissions (GLMM main effect of sex: p=0.1625, β1=0.0057). (c) No significant effect of sex (two-way repeated measures ANOVA effect of sex: p=0.0972, F(1,145)=2.787) or trial (two-way repeated measures ANOVA effect of trial: p=0.6294, F(4,145)=0.6476) on the percent of omissions 1-5 trials following rule shifts during the 10 second response time limit Set Shift schedule. (d) Animals completed four consecutive sessions of the Set Shift task with a three second response time limit. There was no significant effect of sex (two-way repeated measures ANOVA effect of sex: p=0.4876, F(1,116)=0.4849) or session (two-way repeated measures ANOVA effect of session: p=0.8989, F(3,116)=0.1960) on the average percent of omitted trials during this version of the task. (e) Averaging across sessions of this task version revealed no significant effect of sex on the average percent of omissions (GLMM main effect of sex: p=0.1230, β1=0.0113). (f) No significant effect of sex (two-way repeated measures ANOVA effect of sex: p=0.2958, F(1,145)=1.101) or trial (two-way repeated measures ANOVA effect of trial: p=0.9618, F(4,145)=0.1521) on the percent of omissions 1-5 trials following rule shifts during the three second response time limit Set Shift schedule. (g) On average, animals omitted fewer trials during the 10 second response time limit schedule compared to the three second response time limit schedule (paired t-test, p=1.24169e-09, effect size=-19.587). Supp. Figure 11a, 11c, 11d, and 11f depict mean and SEM. Supp. Figure 11b, 11e and 11g depict median (solid line), 1-99th percentile of data in maximum shaded area vertically and kernel density horizontally, and inner hourglass shaded area depicts 25-75% confidence interval of data.

# Supplemental Tables

**Table S1:** Reinforcement Learning Drift Diffusion Model (RLDDM) Parameter Reporting

| **Parameter** | **Probability of Direction (pd)** | **Median** | **Distribution in Region of Practical Equivalence (ROPE)** |
| --- | --- | --- | --- |
| Boundary Separation | 59.35% | 0.0828 | 21.50% |
| Drift Rate | 81.60% | 0.3269 | 14.025% |
| Scaling Bias | 99.05% | 0.9791 | 1.35% |
| Non-Decision Time | 18.30% | -0.3163 | 14.075% |
| Learning Rate | 92.20% | 0.5849 | 7.125% |
| Forgetfulness | 72.75% | 0.2294 | 16.875% |
| Surprise | 48.95% | -0.0110 | 19.275% |
